# A new pterosaur tracksite from the Lower Cretaceous of Wuerho, Junggar Basin, China: inferring the first putative pterosaur trackmaker

**DOI:** 10.7717/peerj.11361

**Published:** 2021-06-01

**Authors:** Yang Li, Xiaolin Wang, Shunxing Jiang

**Affiliations:** 1 Key Laboratory of Vertebrate Evolution and Human Origins, Institute of Vertebrate Paleontology and Paleoanthropology, Chinese Academy of Sciences, Beijing, China; 2 Center for Excellence in Life and Paleoenvironment, Chinese Academy of Sciences, Beijing, China; 3 College of Earth and Planetary Sciences, University of Chinese Academy of Sciences, Beijing, China

**Keywords:** Pterosaur tracks, *Pteraichnus wuerhoensis* isp. nov., Lower Cretaceous, Junggar Basin, Wuerho, Xinjiang

## Abstract

We report the discovery of 114 small pterosaur footprints preserved in a greyish-green fine sandstone slab comprising 57 manus imprints and 57 pes imprints. Due to the chaotic distribution of footprints, the trackways are difficult to recognize. The pes imprints are sub-triangular and enlongate, the metatarsal part is roughly subequal to the digital part. The manus imprints are asymmetrical, longer than wide, and the lengths of digits I–III gradually increase. According to the diagnostic features of the Wuerho small pterosaur tracks, the present set was classified as *Pteraichnus* and is different from the nine reported valid ichnospecies of *Pteraichnus*. We therefore propose a new ichnospecies, *Pteraichnus wuerhoensis* isp. nov. The description is based on the anatomical characteristics (lengths of digits I–IV, length of digital part, length of metatarsal part) extracted from the pes imprints and comparisons with the pes bone fossils of *Noripterus complicidens*. We infer that the footprints were probably left by *N. complicidens* and the total width of the wings was presumably 2–2.3 m. In addition, the high density (365 per square meter) and varied sizes of the Wuerho small pterosaur tracks suggest that many pterosaurs of different ages lived in Huangyangquan Reservoir tracksite 1 area. Thus the trackmakers may have had gregarious behavior.

## Introduction

The study of purported pterosaur footprints began in the 1860s, when Oppel (1862) reported simple straight locomotion traces from the Late Jurassic Solenhofen Limestone in Germany and argued that the tracks were attributable to the pterosaur *Rhamphorhynchus*. However, these traces were later convincingly demonstrated to be the tracks of limulids (Caster, 1941; Malz, 1964). Stokes (1957) reported pterosaur tracks and trackway from the Upper Jurassic Morrison Formation in Apache, Arizona, USA, and named them *Pteraichnus saltwashensis*. However, Padian (1983a, 1983b), and Padian & Olsen (1984) argued that *Pteraichnus* had been made by a small crocodilian. They showed some similarities between the footprints of small crocodilian and *Pteraichnus* trackways. Due to the scarcity of specimens at that time, their reinterpretation was widely accepted. However, with the discovery of a large number of new obvious *Pteraichnus* or *Pteraichnus*-like tracksites in the mid-1990s (Logue, 1994; Lockley et al., 1995; Lockley & Hunt, 1995; Mazin et al., 1995), manus imprints only include the traces of digits I–III (Mazin et al., 1995), and most studies acknowledge that *Pteraichnus* and *Pteraichnus*-like tracks were pterosaurian (Lockley et al., 1995; Unwin, 1996; Mazin et al., 2003). The first convincing non-pterodactyloid pterosaur footprints were reported from the Upper Jurassic Cazals Formation in the pterosaur Beach, Crayssac, France; these were characterized by being pentadactyl (with a well-preserved digit V), with plantigrade to digitigrade pes imprints and tridactyl digitigrade manus imprints with digits anteriorly oriented (Mazin & Pouech, 2020). To date, three pterosaur ichnofamilies, five pterosaur ichnogenera, 15 pterosaur ichnospecies and more than 19 *Pteraichnus* isp., cf. *Pteraichnus*, *Pteraichnus*-like tracks have been reported from at least 77 pterosaur tracksites in 13 countries (Lockley, Harris & Mitchell, 2008; Fiorillo et al., 2009, 2015; Mazin & Padian, 2009; Lee et al., 2010; Hornung & Reich, 2013; Li, 2015; Xing et al., 2016a, 2016b; Ha et al., 2018; Elgh, Pieńkowski & Niedźwiedzki, 2019). The first pterosaur footprints from China were reported by Peng et al. (2004), who named them *P. yanguoxiaensis*. Additional tracksites of pterosaur footprints in China have been discovered, but the preservation conditions have been poor, and the quantities relatively small. There are eight pterosaur tracksites that have been reported in China and all the pterosaur footprints belong to the genus *Pteraichnus* (Li, 2015; Xing et al., 2016a, 2016b). The age of the pterosaur footprints in China is concentrated in the Early Cretaceous and only the pterosaur footprints discovered in Zhejiang and Guangdong provinces are from the Late Cretaceous (Li, 2015; Xing et al., 2016a, 2016b).

The paleontological research in Wuerho region began in the 1960s. In 1963, Jingming Wei of the paleontological Division, Institute of Science, Bureau of Petroleum of Xinjiang, collected a batch of vertebrate fossils in Wuerho in the northwestern Junggar Basin and sent the fossils to the IVPP. Subsequently, Young (1964) identified the fossils as a kind of pterosaur fossils and named them as *Dsungaripterus weii*, which is the earliest named pterosaur fossil in China. Subsequently, a Xinjiang paleontology expedition team was organized by the IVPP in 1964 to conduct further investigations and fossil excavations in the Wuerho region. In addition to collecting abundant pterosaur fossils, turtle, crocodyliform, plesiosaur, and a variety of dinosaur fossils were also discovered, demonstrating that the Wuerho region was a new location abundant in Early Cretaceous vertebrate fossils. This vertebrate group was named the Wuerho Pterosaur Fauna (Young, 1973). Later, when some researchers studied the sedimentary characteristics of the Tugulu group in the Wuerho region, they found fossils of *Psittacosaurus xinjiangensis* (Brinkman et al., 2001), and this enriched the abundance and diversity of the Wuerho Pterosaur Fauna. Due to the relative fragmentation of the fossils, the subsequent research on the Wuerho Pterosaur Fauna mainly focused on the re-study and complemental characters of pterosaurs, dinosaurs and turtles (Norman, Dodson & Osmólska, 1990; Dong, 1992; Sun et al., 1992; Sues, 1997; Lucas, 2002; Rauhut & Xu, 2005; Danilov & Parham, 2007; Lü et al., 2009; Li & Ji, 2010; Hone, Jiang & Xu, 2018; Xu et al., 2018; Chen et al., 2020).

The second large-scale investigation of the Wuerho area began in 2006. In order to compare the Mesozoic vertebrate faunas between the Turpan-Hami Basin and the Junggar Basin, the Hami scientific expedition team (IVPP) has conducted scientific investigations for more than ten years in the Hami and Wuerho regions. In addition to the discovery of a large number of pterosaur and dinosaur fossils in the Wuerho region, a high diversity of vertebrate footprints has also been found (Li, Jiang & Wang, 2020). These include pterosaur, dinosaur, bird and turtle tracks. Li, Jiang & Wang (2020) reported theropod footprints in Huangyangquan Reservoir tracksite that are the largest footprints of *Asianopodus*. During this period, some researchers also reported footprints in Moguicheng Dinosaur and Bizarre Stone Museum specimens, including pterosaur, bird, and dinosaur footprints (He et al., 2013; Xing et al., 2013a, 2013b, 2014). Among the reported three pterosaur footprints were described by Xing et al. (2013a) and He et al. (2013) as belonging to medium to large pterosaur footprints; they were classified as *Pteraichus* isp. He et al. (2013) reported a manus-pes set found in the southeastern margin of Huangyangquan Reservoir. The imprints were dated to the Early Cretaceous, but the specific horizon is unknown. Xing et al. (2013a) reported a single manus imprint in the Wuerho asphaltite tracksite located about 15 km to the east of Huangyangquan Reservoir. Although the specific horizon is also unknown, it is stratigraphically higher than tracksite reported by He et al. (2013) (Xing et al., 2013a). The newly-discovered Wuerho small pterosaur tracks described in this paper were located in the valley on the northwestern side of Huangyangquan Reservoir, a new tracksite that we named Huangyangquan Reservoir tracksite 1. We have no evidence with which to judge whether the newly-discovered footprint layer is the same layer described by He et al. (2013) (because there is no field section photo in the latter paper), but it is not the same layer reported by Xing et al. (2013a) (lower than the asphaltite footprint layer).

The Wuerho region has abundant pterosaur footprints, and this article focuses on the newly- discovered small pterosaur tracks. We conducted specific research on the detailed morphological features, the forward orientations of each pterosaur footprint, and the features of local pterosaur pes bone fossils to infer the probable trackmaker and its possible behavior.

## Materials and Methods

IVPP V 26281.2, which is a greyish green sandstone block (125 cm × 25 cm) with 114 natural casts (convex hyporelief) of small pterosaur tracks was collected from Huangyangquan Reservoir tracksite 1, Wuerho region, northwestern Junggar Basin, Xinjiang, China. The photographs of the overall block and parts of the block were taken, and we then use the CorelDRAW to draw the outlines of the footprints. According to the recent standard protocol for documenting fossil ichnological data proposed by Falkingham et al. (2018), we used photogrammetry and Agisoft PhotoScan Professional to establish three-dimensional models. The 3D Photographic-model and associated data have been uploaded to the MorphoSource (ark:/87602/m4/346640). Length of pes imprint (Lp), width of pes imprint (Wp), length of manus imprint (Lm), width of manus imprint (Wm), length/width (L/W), length of digits I–III or I–IV (LD), divarication angles between digits (DA), length of digital part in pes imprint (D), length of metatarsal part in pes imprint (Me), and forward orientation of each footprint (FO) were measured according to the standards of Pascual-Arribas & Hernández-Medrano (2012) and Hernández-Medrano, Pascual-Arribas & Pérez-Lorente (2017). Statistical analysis was performed using Grapher 12 and IBM SPSS Statistics 24.

The electronic version of this article in Portable Document Format (PDF) will represent a published work according to the International Commission on Zoological Nomenclature (ICZN), and hence the new names contained in the electronic version are effectively published under that Code from the electronic edition alone. This published work and the nomenclatural acts it contains have been registered in ZooBank, the online registration system for the ICZN. The ZooBank LSIDs (Life Science Identifiers) can be resolved and the associated information viewed through any standard web browser by appending the LSID to the prefix http://zoobank.org/. The LSID for this publication is: (urn:lsid:zoobank.org:pub:DF34F23C-8F5A-4CA7-AF42-EB95961E6418). The online version of this work is archived and available from the following digital repositories: PeerJ, PubMed Central and CLOCKSS.

## Results

### Geological setting and stratigraphic sequence

The Wuerho region is located in the northwestern Junggar Basin that belongs to Karamay City, Xinjiang Uygur Autonomous Region, China and is about 100 km away from the urban area of Karamay city. The exposed beds in the Wuerho region are mainly Cretaceous lacustrine sediments (Dong, 1973) that belong to the Tugulu Group (Lower Cretaceous). The Tugulu Group is divided into three Formations from the bottom to top, the Hutubihe Formation, the Shengjinkou Formation, and the Lianmuqin Formation (Zhao, 1980). The Wuerho region lacks the Qingshuihe Formation. The lithology of the Hutubihe Formation is thick or medium-thick greyish-green fine sandstone interbedded with red-brown, light-red mudstone or mudstone lenses and the bottom is medium-thick chartreuse breccia. The fine sandstone also has pillow calcic nodules (Dong, 1973; Zhao, 1980). The lithology of the Shengjinkou Formation is thin or thick greyish-green sandstone interbedded with greyish-yellow, greyish-green mudstone or mudstone lenses. In the middle and upper parts, a large number of calcic nodules (varying in size) are developed. At the top, the main signing bed of white tuffaceous sandstone (approximately 1.5 m) is well developed (Dong, 1973; Zhao, 1980). The lithology of Lianmuqin Formation is thin or thick greyish-green sandstone interbedded with thin red-brown or light-red mudstone. At the top, a large number of ferruginous nodules are developed in the sandstone (Dong, 1973; Zhao, 1980).

**Figure 1:**
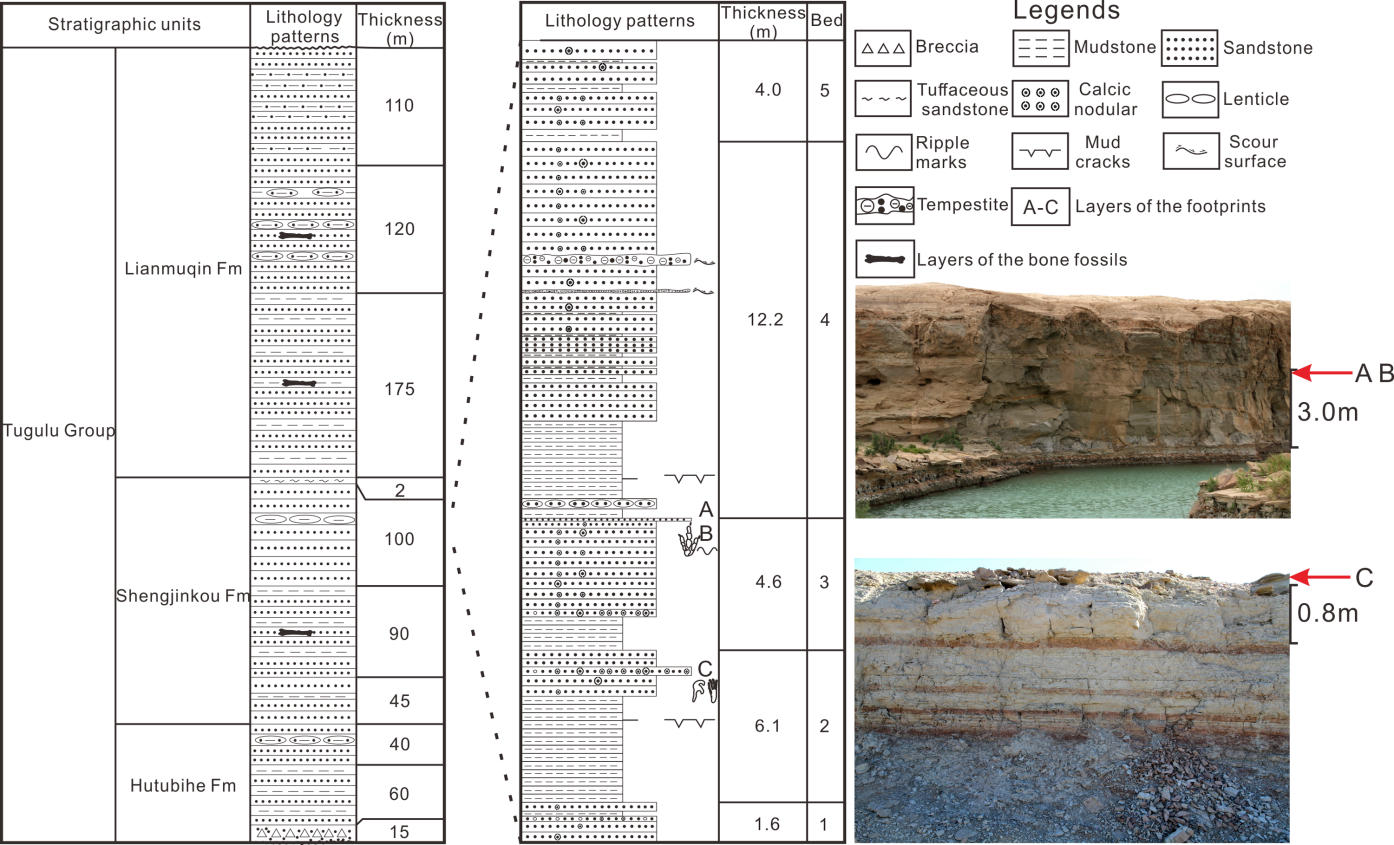
The comprehensive column of Huangyangquan Reservoir tracksite 1 in the Wuerho region. (A–C) represent the layers of the footprints.

The site of Wuerho small pterosaur tracks is located at the west of Wuerho region. The stratigraphic section of the tracksite is divided into five beds and is 28.5 m thick (Fig.1). The stratigraphic sequence is as follows (from top to bottom).

The fifth bed, which is composed of the lower red mudstone and the upper variegated fine sandstone (interbedded with red mudstone, gray mudstone and fine sandstone) has a thickness of 4.0 m and the sandstone is rich in calcic nodules.

The fourth bed, which is composed of lower mudstone and upper greyish fine sandstone, has a thickness of 12.2 m. The mudstone is composed of lower red mudstone and upper greyish mudstone. The red mudstone develops mud cracks structure and the sandstone is interbedded by two layers of tempestite and multiple layers of variegated mudstone. The sandstone also contains abundant spherical and beading calcic nodules varying in size.

The third bed, which consists of greyish mudstone (with red mudstone) in the lower part and greyish-white fine sandstone in the upper part, has a thickness of 4.6 m. The bottom of the sandstone is a greyish-white calcareous fine sandstone layer (parts of the region are coarse sandstone) with a thickness of about 40 cm rich in variously shaped calcic nodules (spherical, dendritic and beading nodules). The top of the sandstone is greyish-green fine sandstone with a thickness of about 6 cm that is rich in *Asianopodus* footprints, invertebrate traces and symmetrical ripples (Fig. 1, footprint layers of A and B) (Li, Jiang & Wang, 2020).

The second bed, which is composed of lower mudstone and upper greyish fine sandstone, has a thickness of 6.1 m. The mudstone consists of lower greyish mudstone and upper variegated mudstone (mainly red, but also mixed with gray mudstone or sandstone). The sandstone is rich in calcic nodules of different sizes. The middle part of the sandstone contains a greyish-white calcareous fine sandstone layer (parts of the region are gravel-bearing sandstone) with a thickness of 40 cm that is abundant with calcic nodules of various sizes (mainly spherical and beading nodules) and small pterosaur footprints (Fig.1, footprint layer C).

The first bed, which is composed of greyish fine sandstone, has a thickness of 1.6 m. The sandstone is rich in calcic nodules of different sizes and the middle part is interbedded with a calcareous gravel-bearing sandstone (roughly 20 cm).

According to the petrological characteristics of the stratigraphic section in Huangyangquan Reservoir tracksite 1 and detailed field surveys, all of the footprint layers are located below the main signing layer of white tuffaceous sandstone. The overall lithology of the section is greyish-green fine sandstone interbedded with greyish mudstone or variegated mudstone and the sandstone is rich in calcic nodules of different sizes. These features are consistent with the lithological characteristics of the sixth bed described by Dong (1973) and Zhao (1980). Therefore, the horizon of the footprint layers belongs to the Shengjinkou Formation (Fig. 1).

### Systematic Ichnology

**Order:** Pterosauria Kaup, 1834

**Suborder:** Pterodactyloidea Plieninger, 1901

**Ichnofamily:** Pteraichnidea Lockley et al., 1995

**Ichonogenus:** *Pteraichnus* Stokes, 1957

Quadruped, elongate, asymmetrical, digitigrade, tridactyl manus imprint; digit I anterior or anterolateral (generally with a claw mark); digit II anterolateral to posterolateral; digit III posterior, digits increasing respectively in length, rounded impression in the medial margin of the well-preserved manus imprint (impression of the fourth metacarpo-phalangeal joint); elongate, subtriangular, plantigrade, tetradactyl pes imprint; middle two digits are longer than the lateral digits; manus imprints on the same axis or more laterally to the pes imprints and the pes imprints anterior to the manus imprints; manus imprints generally as or more deeply impressed than pes imprints.

**Type ichnospecies:** *Pteraichnus wuerhoensis* isp. nov.

**Derivation of the name:** from the locality where the small pterosaur tracks were discovered. The Wuerho region is also the fossil site of Wuerho Pterosaur Fauna.

**Figure 2:**
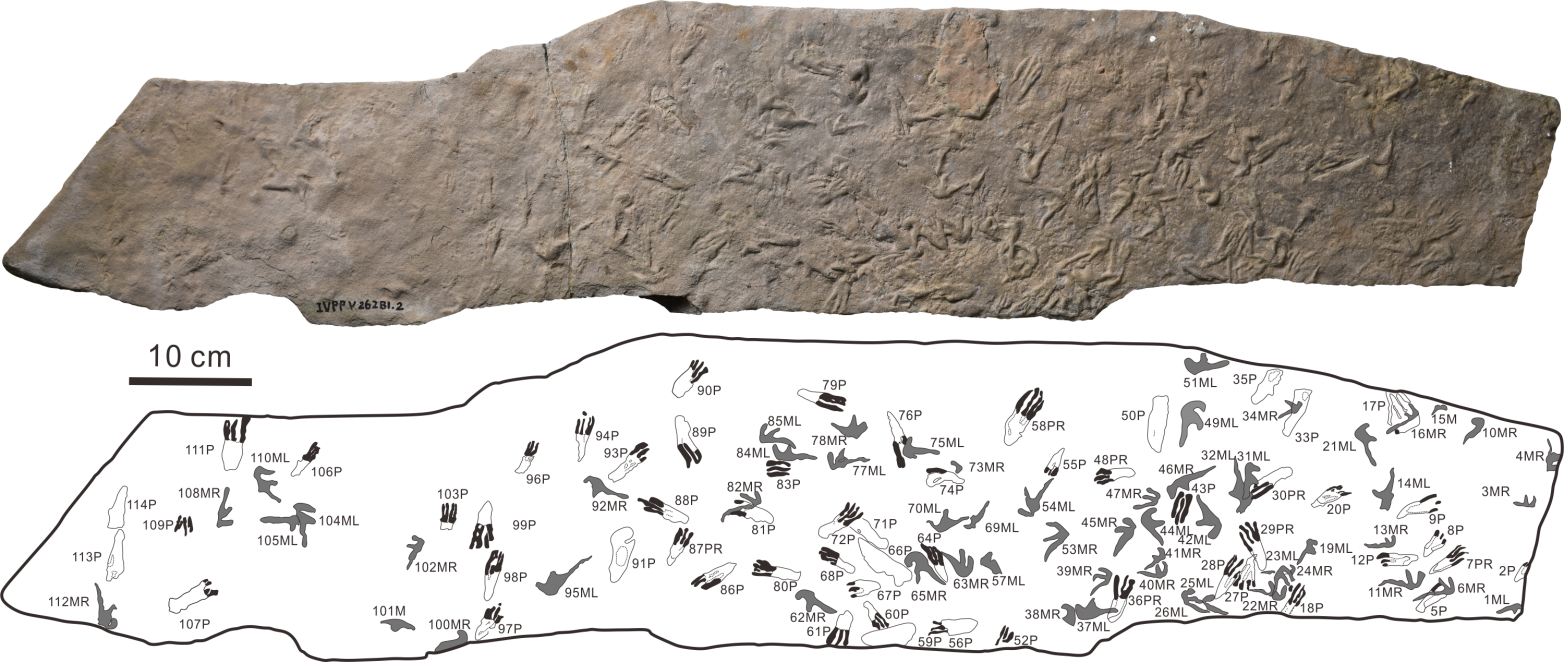
The photograph and outline drawings of *Pteraichnus wuerhoensis* isp. nov. (Photo credit: Wei Gao). Abbreviations: M = Manus imprint; P = Pes imprint; ML = Left imprint of manus; MR = Right imprint of manus; PR = Right imprint of pes.

**Materials: **One slab with 114 natural casts (Fig. 2) on a greyish fine sandstone cataloged as IVPP V 26281.2. The tracks are now stored at IVPP, Beijing, China.

**Holotype:** manus imprint of 45MR, pes imprint of 58PR. The footprints are now stored at IVPP, Beijing, China.

**Paratype:** well preserved manus imprints of 49ML, 104 ML and pes imprints of 7PR and 29PR. The footprints are now stored at IVPP, Beijing, China.

**Referred specimens:** all other footprints (98 footprints, 1ML-6MR, 8P-28P, 30PR-44ML, 46MR-57ML, 59P-103P,105ML-114P) except the holotype and paratype.

**Locality and Horizon:** Shengjinkou Formation (Fig. 1), Tugulu Group, Lower Cretaceous. Huangyangquan Reservoir tracksite 1, Wuerho District, Karamay City, Xinjiang, China.

**Diagnosis:** Quadrupedal tracks, no tail trace; manus imprints are strongly asymmetrical, small-sized, roughly 3.40 cm in length, and 1.59 cm in width, tridactyl, longer than wide, with an average Lm/Wm ratio of 2.14. Digit I is the shortest (roughly 1.31 cm), generally laterally or posterolaterally oriented straight; digit II is intermediate in length (roughly 1.90 cm), posterolaterally oriented, The crescent-shaped digit III is the longest (roughly 3.15 cm), posteriorly oriented with a distal curvature toward the medial side. The average divarication of digit II and digit III is approximately 1.74 times the average divarication between digit I and digit II (33.5°). The pes imprints are elongate and fully plantigrade, tetradactyl, sub-triangular shaped, small-sized (roughly 4.02 cm in length, 1.46 cm in width), with an average Lp/Wp ratio of 2.75. The digit I imprint is the shortest and the other three digits are roughly subequal in length (average length of digits I–IV are 1.66–2.16–2.06–1.97 cm). The metatarsal part is narrow and elongate, roughly subequal to the digital part. The interdigital angle between digits I and IV is about 14.6°.

**Description:**

**Figure 3:**
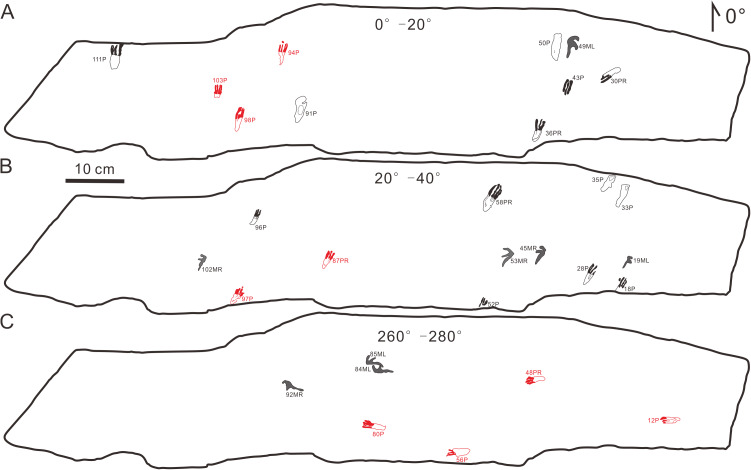
Some possible but uncertain trackways of the same size and forward orientation footprints (the red color possibly represents the same trackway). (A) The tracks in forward orientations of 0°–20°, (B) the tracks in forward orientations of 20°–40°, (C) The tracks in forward orientations of 260°–280°. Abbreviations: P = Pes imprint; ML = Left imprint of manus; MR = Right imprint of manus; PR = Right imprint of pes.

**Figure 4:**
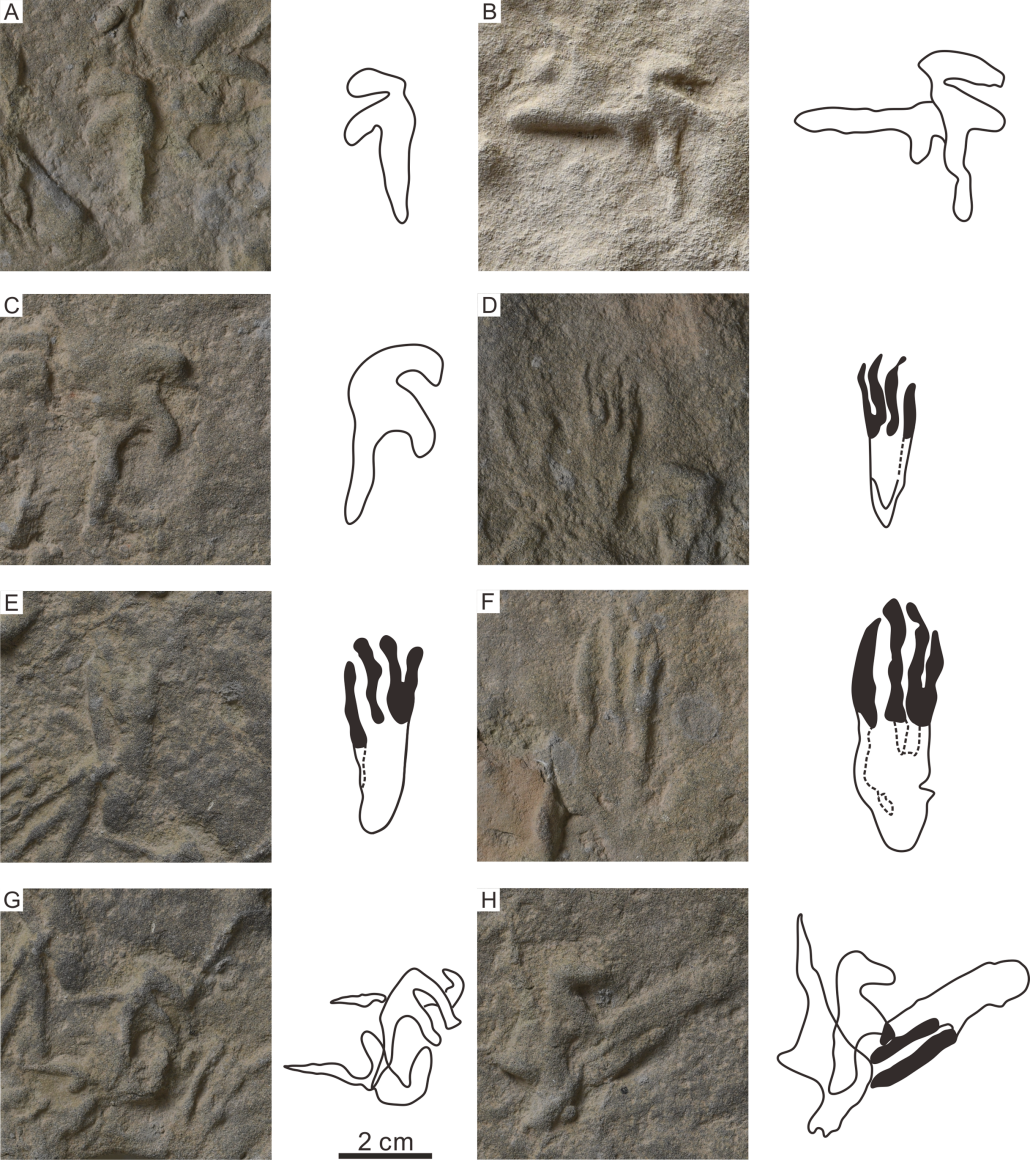
The photographs and outline drawings of the holotype, paratype, and overlapping pterosaur footprints of *Pteraichnus wuerhoensis* isp. nov. in Huangyangquan Reservoir tracksite 1. (A) 45MR, holotype, digit I points to lateral orientation; (B) 104ML, paratype, digit I points to posterolateral orientation; (C) 49ML, paratype, with a little curved digit I; (D)–(E) 7PR, 29PR, paratype, with the shortest digit I and subequal length of other digits; (F) 58PR, holotype, with the shortest digit I and subequal length of other digits; (G) 22MR, 23ML, 24MR, overstepping imprints of three manus imprints; (H) 30PR, 31ML, 32ML, overstepping imprints of two manus imprints and one pes imprint. Abbreviations: P = Pes imprint; ML = Left imprint of manus; MR = Right imprint of manus; PR = Right imprint of pes.

The footprints (57 manus imprints and 57 pes imprints) are located on one slab (125 cm × 25 cm), with symmetric, functionally tetradactyl, fully plantigrade pes impressions and asymmetric, tridactyl, digitigrade manus impressions (Fig. 2). The distribution of the tracks is disordered. In order to find the trackways as far as possible, we computed statistics on the forward orientations of the 114 tracks (Fig. 3, assuming that the orientation perpendicular to the long axis of the slab is 0°) We found that some of the tracks that may have been on the same trackway were uncertain (Fig. 3). For example, according to the forward orientations and sizes of the Wuerho small pterosaur tracks, the following footprints may well be on the same trackway (the same sizes and forward orientations). The tracks of 94P, 98P, and 103P may be on the same trackway (Fig. 3A). Similarly, the 97P and 87PR could also be potentially on the same trackway (Fig. 3B). The tracks 12P, 48PR, 56P and 80P may also belong to the same trackway (the same sizes and orientations). However, there are no manus imprints or other tracks that may form a consecutive trackway, so it is impossible to identify whether they are on the same trackway (Figs. 3A–3C). The footprints are very close to each other, and there are many overlapping footprints (Fig. 2). All tracks are similar in shape (Figs. 2, 4A–4H).

The pes footprints are elongate and fully plantigrade, small-sized (roughly 4.02 cm in length, 1.46 cm in width), with an average Lp/Wp ratio of 2.75 (Table 1). Most footprints are sub-triangular shaped (Figs. 4D–4F), while some are rectangular (possibly related to different preservation conditions, Fig. 2). The tracks show four functional digits and indistinct digital pads. The digit I imprint is the shortest, and the other three digits are roughly subequal in length (the medial two digits are slightly longer than digit IV; average lengths of digits I–IV are 1.66, 2.16, 2.06, and 1.97 cm, respectively). Only one footprint (7PR) retains a clear claw mark on the anterior part of digit III. The claw mark is curved laterally and gradually distended toward the distal end. The metatarsal area is narrow and elongate and is roughly subequal to the digital area (Table 1, average D/Me = 1.00). Most heel imprints are very poorly marked and slightly lighter than the digital prints. The interdigital angle between digits I and IV is about 5.4°–35.7° (Table 1, average 14.6°). There is no preservation of impressions formed by the fifth metatarsal and digit V.

**Table 1:** The measurement parameters of *Pteraichnus wuerhoensis* isp. nov. and pes bone fossils of *N. complicidens*. Abbreviations: M = Manus imprint; P = Pes imprint; ML = Left imprint of manus; MR = Right imprint of manus; PR = Right imprint of pes; FL = Footprint length; FW = Footprint width; L/W = Length/Width; LD = Lengths of digits I–III or I–IV; DA = Divarication angels between digits; D = Length of digital part in pes imprint; Me = Length of metatarsal part in pes imprint; FO = Forward orientation of each footprint.

Footprints	FL	FW	L/W	LD	DA			D(cm)	Me(cm)	D/Me	FO	M
				I–II–III/I–II–III–IV	I–II	II–III	I–III/I–IV					Digit I position
1ML	2.30	0.85	2.71	0.45?–0.90–2.00	48.6°	65.0°	113.6°				69.1°	laterally
2P	2.0?										42.0°	
3MR	1.90	1.00	1.90	1.10–1.40–1.80	24.3°	79.8°	104.1°				96.7°	laterally
4MR	2.85	1.24	2.30	1.04–1.71–2.80	6.5°	80.8°	87.3°				174.8°	laterally
5P	3.06?	1.34?	2.28?				19.5°	0.88?	2.18?	0.40?	40.8°	
6MR	3.83	1.66	2.31	1.30–1.74–3.68	27.2°	58.5°	85.7°				73.5°	laterally
7PR	3.87	1.28	3.02	1.22–1.74–1.58–1.60			17.6°	1.90	1.97	0.96	64.5°	
8P	2.62?	1.35	1.94?				22.2°	0.56?	2.06	0.27?	42.9°	
9P	4.03?	1.43	2.82?				15.4°	1.74?	2.29	0.76?	72.0°	
10MR	2.72?	1.15?	2.66?			47.7°					47.9°	
11MR	3.77	1.86	2.03	1.33–1.81–3.32	42.0°	56.6°	98.6°				102.8°	anterolaterally
12P	3.56?	1.61	2.21?				11.8°	1.04?	2.52	0.41?	266.5°	
13MR	2.68	1.07	2.50	0.87–1.01–2.56	31.7°	56.7°	88.4°				85.7°	laterally
14ML		1.86?				69.1°					193.4°	
15M												
16MR	3.30	1.30?	2.54?	1.17–1.19?–3.28	23.3°	60.1°	83.4°				58.1°	laterally
17P		2.71?					37.0°?				155.1°	
18P	3.12?	0.99	3.15?				2.7°?	2.07			33.1°	
19ML	2.58	1.21	2.13	0.83–1.39–2.44	23.1°	81.7°	104.8°				31.3°	laterally
20P	3.49	1.22	2.86				19.9°	1.75?	1.74?	1.01?	59.6°	
21ML	3.66	1.53	2.39	1.38–1.95–3.41	50.5°	45.6°	96.1°				338.5°	laterally
22MR	3.19	1.41	2.26	1.17–1.91–3.12	44.6°	41.5°	86.1°				106.4°	laterally
23ML	2.87	1.75	1.64	1.62–1.78–2.83	5.7°	50.0°	55.7°				19.2°?	posterolaterally
24MR	2.95	0.90?	3.28?	0.87–?–2.75			102.9°				90.6°	laterally
25ML						47.2°					289.7°	
26ML	2.95	1.41	2.09	1.21–1.95–2.89	18.8°	33.8°	52.6°				298.1°?	posterolaterally
27P	3.35	1.89	1.77				35.7°	0.99?	2.36	0.42?	198.7°	
28P	4.31	1.25	3.45				16.2°	2.12	2.19	0.97	31.3°	
29PR	4.39	1.58	2.78	1.67–2.00–1.91–1.95			16.7°	2.25	2.14	1.05	334.8°	
30PR	4.28	1.27	3.37	?–?–?–2.19			5.4°	2.18	2.10	1.04	240.4°	
31ML	3.24	1.29	2.51	1.17–1.97–3.17	19.9°	59.9°	79.8°				9.2°	posterolaterally
32ML	5.00	2.04	2.45	1.61–2.08–4.09	73.7°	65.1°	138.8°				164.9°	anterolaterally
33P	4.49	1.38	3.25				14.4°				24.9°	
34MR	2.44?	1.43?	1.71?		123.7°?	29.2°	152.9°?				61.0°	
35P	3.74	1.52	2.46				6.6°				38.4°	
36PR	4.25	1.82	2.34	?–?–?–1.94			8.3°	2.14	2.11	1.01	14.9°	
37ML	3.71	1.79	2.07	1.24–2.19–3.45	45.9°	68.3°	114.2°				263.4°	
38MR				1.17–?–?								
39MR	2.75	1.55	1.77	1.27–1.72–2.70	13.9°	47.1°	61.0°				42.3°?	laterally
40MR	2.59	1.48	1.75	1.26–1.18–2.40	21.1°	66.3°	87.4°				102.9°	laterally
41MR	2.31	1.12	2.06	1.07–1.13–2.02	48.8°	66.5°	115.3°				58.5°	laterally
42ML	4.00	1.90	2.11	1.46–2.18–3.40	53.4°	65.2°	118.6°				151.9°	laterally
43P											18.0°	
44ML	3.26	1.79	1.82	1.64–2.06–2.93	27.7°	59.3°	87.0°				320.6°	laterally
45MR	3.51	1.60	2.19	1.30–1.88–3.19	42.5°	53.2°	95.7°				31.1°	laterally
46MR	4.71	1.40	3.36	1.12–1.88–4.54	36.4°	77.1°	113.5°				244.6°	laterally
47MR	2.50	1.67	1.50	1.30–1.54–2.49	12.6°	25.5°	38.1°				75.0°?	laterally
48PR	3.64	1.41	2.58	?–?–1.31–1.40			13.9°	1.79	1.85	0.97	261.5°	
49ML	4.25	2.17	1.96	1.55–2.26–3.82	39.6°	44.6°	84.2°				10.6°	laterally
50P	4.88	1.60	3.05				9.4°				7.9°	
51ML	3.82	1.84	2.08	1.54–2.00–3.24	39.5°	74.7°	114.2°				255.8°	anterolaterally
52P											28.0°	
53MR	3.35	2.01	1.67	1.98–2.29–3.17	53.6°	34.2°	87.8°				39.2°	laterally
54ML	4.26	2.06	2.07	1.40–2.55–3.68	49.6°	49.2°	98.8°				215.5°	laterally
55P	2.68	1.15	2.33				14.7°	0.92?	1.76	0.52?	209.9°	
56P	4.30	1.41	3.05				7.8°	1.74?	2.56	0.68?	261.6°	
57ML												laterally
58PR	5.66	1.93	2.93	2.10–2.73–2.70–2.37			9.4°	2.82	2.84	0.99	32.0°	
59P	4.77	1.87	2.55				13.2°				76.5°	
60P	3.59	1.61	2.23				19.1°	1.81	1.78	1.02	144.8°	
61P	3.31	1.86	1.78				17.3°	1.62?	1.69	0.96?	199.0°	
62MR	3.71	1.68	2.21	1.02–1.92–3.40	38.7°	62.3°	101.0°				300.1°	laterally
63MR	3.20	1.79	1.79	1.74–2.40–3.11	9.8°	44.9°	54.7°				126.8°?	posterolaterally
64P	3.90	1.32	2.95				13.7°	2.40?	1.50	1.60?	336.9°	
65MR	4.09	2.03	2.01	1.50–2.76–3.89	37.9°	49.5°	87.4°				309.5°	laterally
66P	5.71	1.79	3.19				21.8°				128.1°	
67P	2.73?	1.50	1.82?				24.3°	0.98?	1.78	0.55?	257.5°	
68P	3.46	1.12	3.09				11.1°	1.71	1.75	0.98	297.0°	
69ML	3.00	0.79?	3.80?	0.67?–0.98?–2.73			131.8°				214.7°	
70ML	3.48	1.63	2.13	1.39–2.07–2.92	53.6°	63.5°	117.1°				242.7°	
71P	3.52?	1.39	2.53?				13.2°				308.4°	
72P	4.19	1.61	2.60				10.1°	2.10	2.09	1.00	52.3°	
73MR												
74P	3.32	1.59	2.09				20.4°	1.66?	1.66	1.00?	278.0°?	
75ML						78.5°					277.2°?	
76P	4.97	1.41	3.52				17.4°	2.31?	2.66	1.15?	158.3°	
77ML	3.62	1.56	2.32	1.52–2.12–3.35	31.0°	57.0°	88.0°				259.5°	laterally
78MR	2.87?	1.93?	1.49?	1.58?–2.06–2.56?	47.1°	65.2°	112.3°				117.1°	
79P	4.23	1.32	3.20				17.1°	2.38?	1.85	1.29?	107.9°	
80P	4.06	1.43	2.84				11.1°	2.01	2.05	0.98	279.8°	
81P	3.59?	1.33	2.70?				14.3°	1.33?	2.26	0.59?	291.9°	
82MR	3.78	1.89	2.00	1.05–1.93–3.64	42.4°	48.4°	90.8°				68.9°	laterally
83P											90°	
84ML	3.83	1.32?	2.90?	1.29–1.50?–3.33	36.0°	79.3°	115.3°				268.6°	anterolaterally
85ML	3.19	1.67	1.91	1.60–1.97–3.00	32.6°	48.4°	81.0°				269.6°	laterally
86P	3.95	1.35	2.93				14.9°	2.10?	1.85	1.14?	232.3°	
87PR	3.62	1.41	2.57	?–?–?–1.50			14.2°	1.67?	1.95	0.86?	26.7°	
88P	4.05	1.30	3.12				11.3°	2.05?	2.00	1.03?	291.3°	
89P	4.15	1.55	2.68				10.5°	2.00?	2.15	0.93?	156.5°?	
90P	3.85	1.40	2.75				9.4°	1.94	1.91	1.02	42.4°	
91P	4.70	2.05	2.29				18.2°				14.0°	
92MR	4.00	1.70	2.35	0.80–1.75–3.70	31.1°	85.2°	116.3°				279°	laterally
93P	4.05	1.30	3.12				8.9°	1.50?	2.55	0.59?	52.1°	
94P	4.35	1.40	3.11				18.0°	2.20	2.15	1.02	7.2°	
95ML	5.15	1.90?	2.71?		67.6°	65.9°	133.5°				229.3°	
96P	3.00	1.20	2.50				6.2°	1.40?	1.60	0.88?	27.2°	
97P	3.40	1.40	2.43				19.4°	1.70?	1.70	1.00?	35.1°	
98P	4.15	1.30	3.19				12.9°	2.10	2.05	1.02	10.8°	
99P	3.95	1.65	2.39				22.9°	1.95	2.00	0.98	190.8°	
100MR				1.10?–?–?								laterally
101M												
102MR	2.75	1.30	2.12	1.00–1.60–2.60	10.3°	78.4°	88.7°				29.2°	laterally
103P								1.65			8.5°	
104ML	3.50	1.45	2.41	1.30–1.95–3.30	2.8°	66.2°	69.0°				355.7°	posterolaterally
105ML						87.1°					90.3°	
106P	3.30	0.95	3.47				6.5°	1.80?	1.50	1.20?	41.3°	
107P	4.00?	1.55	2.58?				8.0°				63.2°	
108MR	3.40	1.30	2.62	1.15–2.10–3.20	43.7°	34.8°	78.5°				193.0°	posterolaterally
109P											184°	
110ML	3.60	1.80	2.00	1.80–2.30–3.30	3.0°	38.1°	41.1°				315.2°	posterolaterally
111P	4.40?	2.15	2.05?				12.6°	1.90?	2.50	0.76	7.8°	
112MR	3.75	1.40	2.68	1.0–1.85–3.30	27.6°	55.4°	83.0°				165.0°	posterolaterally
113P	3.95	1.30	3.04				16.2°				188.9°	
114P	3.30	1.25	2.64				17.3°				191.9°	
Average M	3.40	1.59	2.14	1.31–1.90–3.15	33.5°	58.3°	91.8°					
P	4.02	1.46	2.75	1.66–2.16–2.06–1.97			14.6°	2.07	2.07	1.00		
Data of pes bone	5.98			1.90–2.45–2.55–2.40				2.98	3.00	0.99		

The strongly asymmetrical manus imprints (Figs. 2, 4A–4C) are also small-sized (Table 1, roughly 3.40 cm in length, 1.59 cm in width), tridactyl, longer than wide, with an average Lm/Wm ratio of 2.14 (Table 1). The manus imprints have three digital impressions but lack digit pad impressions and claw marks. In general, digit I is the shortest (roughly 1.31 cm), generally laterally or posterolaterally oriented (of 42 well-preserved manus imprints, 38 are lateral or posterolateral, Table 1) and nearly straight, while some digit I impressions of the manus imprints are slightly curved (Fig. 4C). Digit II is intermediate in length (roughly 1.90 cm), 1.45 times the size of digit I, posterolaterally oriented and nearly straight. The crescent-shaped digit III is the longest (roughly 3.15 cm), 2.4 times the size of digit I, posteriorly oriented with a distal curvature toward the medial side. The total divarication of digit I–III impressions ranges from 38.1° to 138.8° (Table 1, average 91.8°). Average divarication of digit II and digit III is 58.3°, which is approximately 1.74 times the average divarication between digit I and digit II (33.5°). There are no metacarpophalangeal joint or digit IV impressions.

## Discussion

### Comparison with the pterosaur footprints

More than 77 pterosaur tracksites have been reported from East Asia, North America, South America, Europe and North Africa (13 countries) (Lockley, Harris & Mitchell, 2008; Fiorillo et al., 2009, 2015; Lee et al., 2010; Hornung & Reich, 2013; Li, 2015; Xing et al., 2016a, 2016b; Ha et al., 2018). Since the first confirmed pterosaur footprints were discovered along the southwestern coastline of South Korea (Lockley et al., 1997), more than 30 pterosaur tracksites have been reported from the Early Cretaceous to the Late Cretaceous throughout Asia (Korea, China and Japan) (Lockley, Harris & Mitchell, 2008; Lee et al., 2010; Li, 2015; Xing et al., 2016a, 2016b; Ha et al., 2018). To date, we recognize three pterosaur ichnofamilies, five pterosaur ichnogenera and 15 pterosaur ichnospecies as valid (Lockley et al., 1995; Li, 2015; Lockley, Harris & Mitchell, 2008; Sánchez-Hernández, Przewieslik & Benton, 2009; Masrour et al., 2018; Mazin & Pouech, 2020). These are Ramphichnidae (*Ramphichnus crayssacensis*, *R. pereiraensis*, and *R. lafaurii*) (Mazin & Pouech, 2020), Agadirichnidae (*Agadirichnus Elegans and Haenamichnus uhangriensis*) (Hwang et al., 2002; Masrour et al., 2018), and Pteraichnidae (*Purbeckopus pentadactylus*, *Pteraichnus stokesi*, *P. saltwashensis*, *P. parvus*, *P. longipodus*, *P. koreanensis*, *P. palacieisaenzi*, *P. dongyangensis*, *P. yanguoxiaensis*, and *P. nipponensis*) (Stokes, 1957; Lockley et al., 1995; Wright et al., 1997; Peng et al., 2004; Sánchez-Hernández, Przewieslik & Benton, 2009; Lee et al., 2010; Chen et al., 2013; Pascual-Arribas et al., 2014).

**Figure 5:**
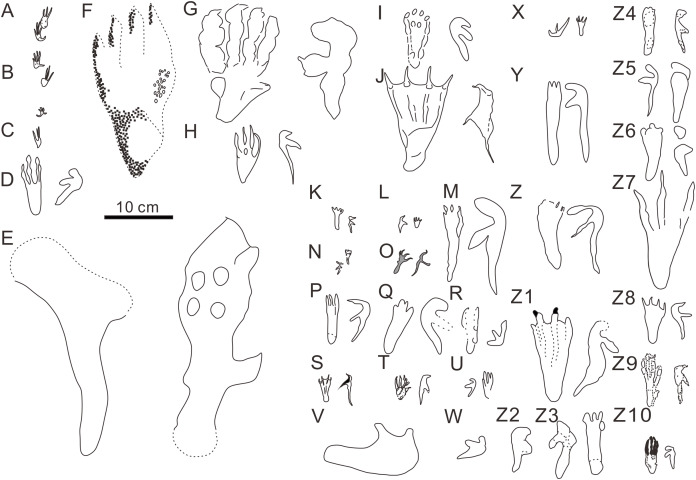
Comparison of reported pterosaur footprints found worldwide. (A) *Ramphichnus* *crayssacensis*, holotype, Lower Tithonian, Upper Jurassic, Crayssac, France (Mazin & Pouech, 2020); (B) *Ramphichnus* *pereiraensis*, holotype, Lower Tithonian, Upper Jurassic, Crayssac, France (Mazin & Pouech, 2020); (C) *Ramphichnus* *lafaurii*, holotype, Lower Tithonian, Upper Jurassic, Crayssac, France (Mazin & Pouech, 2020); (D) *Agadirichnus* *elegans*, holotype, Maastrichtian, Upper Cretaceous, Agadir, Morocco (Masrour et al., 2018); (E) *Haenamichnus* *uhangriensis*, holotype, Uhangri Formation, Upper Cretaceous, Jeollanam Province, Korea (Hwang et al., 2002); (F) *Haenamichnus* *gainensis*, holotype, Aptian-Albian, Upper Lower Cretaceous, Gyeongsang Province, Korea (Kim et al., 2012); (G) *Purbeckopus pentadactylus*, holotype, Lower Cretaceous, Dorset, England (Delair, 1963); (H) *Pteraichnus* *saltwashensis*, holotype, Morrison Formation, Late Jurassic, Arizona, America (Stokes, 1957); (I) *Pteraichnus* *stokesi*, holotype, Sundance Formation, Upper Jurassic, Wyoming, America (Lockley et al., 1995); (J) *Pteraichnus* *palacieisaenzi*, pes from the holotype, manus from the paratype, Middle Berriasian, Lower Cretaceous, Soria, Spain (Pascual-Arribas et al., 2014); (K) *Pteraichnus* *longipodus*, holotype, Berriasian, Lower Cretaceous, Soria Province, Spain (Fuentes-Vidarte et al., 2004); (L) *Pteraichnus parvus*, holotype, Berriasian, Lower Cretaceous, Soria Province, Spain ( Meijide-Calvo, 2001); (M) *Pteraichnus* *yanguoxiaensis*, holotype, Lower Cretaceous, Gansu, China (Peng et al., 2004); (N) *Pteraichnus* *koreanensis*, holotype, Lower Cretaceous, Hadong County, South Korea (Lee et al., 2009); (O) *Pteraichnus* *nipponensis*, holotype, Lower Cretaceous, Fukui Prefecture, Japan (Lee et al., 2010); (P) *Pteraichnus* *dongyangensis*, holotype, Lower Cretaceous, Zhejiang, China (Chen et al., 2013); (Q) cf. *Pteraichnus*, Lower or Later Cretaceous, Neuquén State, Argentina (Calvo & Lockley, 2001); (R) *Pteraichnus*-like, Maastrichtian, Upper Cretaceous, Utah, America (Lockley, 1999); (S) *Pteraichnus* isp., Lower Tithonian, Upper Jurassic, Crayssac tracksite, France (Mazin et al., 2003); (T) *Pteraichnus* isp., Early Kimmeridgian, Upper Jurassic, Wierzbica, Poland (Pieńkowski & Niedźwiedzki, 2005); (U) cf. *Pteraichnus*; Upper Jurassic, Utah, America (Mickelson et al., 2004); (V) *Pteraichnus* isp., Lower Cantwell Formation, Upper Cretaceous, Alaska, America (Fiorillo et al., 2009); (W) *Pteraichnus* isp., Lower Cantwell Formation, Upper Cretaceous, Alaska, America (Fiorillo et al., 2015); (X) cf. *Pteraichnus*, Maastrichtian, Upper Cretaceous, Agadir, Morocco (Masrour et al., 2017); (Y) *Pteraichnus* isp., Aptian-Albian, Lower Cretaceous, southern coast of Korea (Kim et al., 2006); (Z) *Pteraichnus* isp., Lower Cretaceous, Ulsan, Korea (Ha et al., 2018); (Z1) *Pteraichnus* isp., Lower Cretaceous, Huangyangquan tracksite, Wuerho, Xinjiang, China (He et al., 2013); (Z2) *Pteraichnus* isp., Lower Cretaceous, Wuerho, Xinjiang, China (Xing et al., 2013a); (Z3) *Pteraichnus* isp., Lower Cretaceous, Chongqing, China (Xing et al., 2013c); (Z4) *Pteraichnus* isp., Lower Cretaceous, Jimo city, Shandong Province, China (Xing et al., 2012); (Z5) *Pteraichnus* isp., Lower Cretaceous, Zhaojue County, Sichuan Province, China (Xing et al., 2015); (Z6) *Pteraichnus* isp., Lower Cretaceous, Gulin County, Sichuan, China (Xing et al., 2016b); (Z7) *Pteraichnus* isp., Upper Cretaceous, Nanxiong, Guangdong, China (Xing et al., 2016a); (Z8) pterosaur landing tracks, Upper Jurassic, Crayssac, France (Mazin & Padian, 2009); (Z9) *Pteraichnus* isp., Upper Jurassic, Radom, Poland (Elgh, Pieńkowski & Niedźwiedzki, 2019); (Z10) *Pteraichnus wuerhoensis* isp. nov., Lower Cretaceous, Wuerho, China.

The Ichnofamily Rhamphichnidae (Mazin & Pouech, 2020) comprising non-pterodactyloid pterosaur trackway reported from the Upper Jurassic (Cazals Formation) in the Pterosaur Beach, Crayssac, France, is characterized by pentadactyl (with well-preserved digit V) plantigrade to digitigrade pes imprints and tridactyl digitigrade manus imprints with digits anteriorly orientated (Figs. 5A–5C). The manus trackway is wider than, or equal to the pes trackway. There is no tail trail. Several features of Rhamphichnidae are unparalleled and are quite different from the Wuerho small pterosaur tracks and almost all of the reported pterosaur footprints (i.e., elongate and fully plantigrade pes imprints, strongly asymmetrical manus imprints, and the manus trackway being wider than the pes trackway).

Ichnofamily Agadirichnidae (Masrour et al., 2018), was defined by large-sized footprints (pes imprints ranging from 77 to 170 mm for *Agadirichnus* and reaching 340 mm for *Haenamichnus*), longer and slender tetradactyl, plantigrade pes imprints, generally with narrow and rounded pes heels and rather massive tridactyl manus imprints (Masrour et al., 2018; Mazin & Pouech, 2020). To date, there are two ichnogenera and two ichnospecies are attributed to this family: *Agadirichnus* (Ambroggi & De Lapparent, 1954), with the ichnospecies *A. elegans* (Ambroggi & De Lapparent, 1954), and *Haenamichnus* (Hwang et al., 2002), with the single ichnospecies *H. uhangriensis* (Hwang et al., 2002). *A. elegans*, was first described from Upper Cretaceous (Maastrichtian, Tagragra Formation) by Ambroggi & De Lapparent (1954), but the original specimens are assumed to be lost, and this is assessed as an invalid ichnotaxon (Sánchez-Hernández, Przewieslik & Benton, 2009; Lockley MG, Harris JD, in press). However, Masrour et al. (2018) rediscovered *A. elegans* at the same tracksite, and the tracks are quite different from the small-sized pterosaur tracks in the Wuerho region in size and morphology. The pterosaur tracks from the Wuerho region are approximately half size of *A. elegans* (Table 2). The pes imprint of *A. elegans* has rounded heels and subparallel edges that are quite different from the small pterosaur tracks in the Wuerho region (Fig. 5D) (pes imprints are sub-triangular). The manus imprint of *A. elegans* has a short and rounded digit I (Fig. 5D) that is different from the well-preserved manus imprints from the Wuerho region. The digit I imprints of Wuerho small pterosaur tracks are often oriented laterally or posterolaterally so that the divarication between digits I and II is much smaller than in *A. elegans*. *H. uhangriensis* was reported from the Upper Cretaceous (Uhangri Formation) and is also quite different from the Wuerho small pterosaur footprints in morphology and size. The greatest difference between the *H. uhangriensis* and Wuerho pterosaur footprints is in size; the former is approximately nine times the size of the latter (Table 2). The length/width of *H. uhangriensis* pes imprints is 3.33, larger than the Wuerho small pterosaur tracks (2.75). The pes imprint of *H. uhangriensis* has a narrow, rounded heel and digit V impression (Fig. 5E), also different from the Wuerho small pterosaur tracks (the shape of the pes imprints is sub-triangular, and there are no digit V imprints). There is another fossil of *H. gainensis* from the Lower Cretaceous (Haman Formation) that was considered to be pterosaur footprints (Fig. 5F) (Kim et al., 2012), but more recent studies attributed it to bipedal crocodylomorph tracks (Kim et al., 2020).

**Table 2:** Comparative data for the 15 reported ichnospecies and *Pteraichnus* isp. reported from Wuerho region. Abbreviations: Lp = Length of pes imprint; Wp = Width of pes imprint; Lm = Length of manus imprint; Wm = Width of manus imprint; D = Length of digital part in pes imprint; Me = Length of metatarsal part in pes imprint.

Ichnospecies	Horizon	Lp × Wp (cm)	Lm × Wm (cm)	Divarication angles (manus)		D × Me (cm)	Lp/Wp	D/Me
				I–II	II–III			
*Ramphichnus crayssacensis*	Cazals Fm Upper Jurassic	2.91 × 0.52	3.13 × 0.55	14.80°	22.50°	2.47 × 0.44	5.60	5.61
*R. pereiraensis*	Cazals Fm Upper Jurassic	2.40 × 1.73	2.40 × 0.96	6.40°	17.20°	1.71 × 0.69	1.39	2.48
*R. lafaurii*	Cazals Fm Upper Jurassic	1.68 × 1.45	2.17 × 0.74	7.00°	12.00°		1.16	
*Purbeckopus pentadactylus*	Purbeck Limestone Fm Lower Cretaceous	18.70 × 9.80	14.00 × 8.50	22.90°	79.00°	11.82 × 6.88	1.91	1.72
*Agadirichnus elegans*	Tagragra Formation Upper Cretaceous	9.16 × 3.13	6.21 × 3.09	64.60°	34.40°	4.67 × 4.49	2.93	1.04
*Haenamichnus uhangriensis*	Uhangri Formation Upper Cretaceous	35.0 × 10.50	33.00 × 11.00				3.33	
*Pteraichnus saltwashensis*	Morrison Fm Upper Jurassic	8.76 × 4.33	8.20 × 3.15	43.40°	48.10°	3.71 × 5.05	2.02	0.73
*Pteraichnus stokesi*	Sundance Fm Upper Jurassic	9.00 × 4.10	7.00 × 3.40	13.70°	31.00°	4.48 × 4.52	2.20	0.99
*Pteraichnus parvus*	Huérteles Allo Fm Lower Cretaceous	1.53 × 1.14	2.46 × 1.20	64.00°	58.00°	0.44 × 1.09	1.34	0.40
*Pteraichnus longipodus*	Huérteles Allo Fm Lower Cretaceous	3.40 × 1.74	2.45 × 1.67	47.00°	49.00°		1.95	0.22
*Pteraichnus koreanensis*	Hasandong Fm Lower Cretaceous	2.57 × 1.28	2.56 × 1.23	68.50°	47.60°	0.81 × 1.76	2.01	0.46
*Pteraichnus palacieisaenzi*	Huérteles Allo Fm Lower Cretaceous	15.34 × 11.9	13.02 × 4.85	75.90°	56.20°	8.67 × 6.67	1.29	1.30
*Pteraichnus dongyangensis*	Jinhua Fm Lower Cretaceous	9.00 × 3.00	6.50 × 4.00	52.00°	29.00°		3.00	
*Pteraichnus nipponensis*	Kitadani Fm Lower Cretaceous	1.94 × 1.05	2.01 × 0.88	83.18°	81.70°	0.47 × 1.23	1.85	0.38
*Pteraichnus yanguoxiaensis*	Hekou Fm Lower Cretaceous	12.30 × 3.60	12.20 × 4.80	60.70°	53.90°		3.42	
*Pteraichnus* isp.	Tugulu Group Lower Cretaceous		6.70 × 2.90	62.70°	56.00°			
*Pteraichnus* isp.	Tugulu Group Lower Cretaceous	14.00 × 6.00	12.30 × 5.20	58.00°	70.00°	6.07 × 7.93	2.33	0.77

**Note:**

The values obtained directly from the original papers or the outline drawings in the papers.

**Figure 6:**
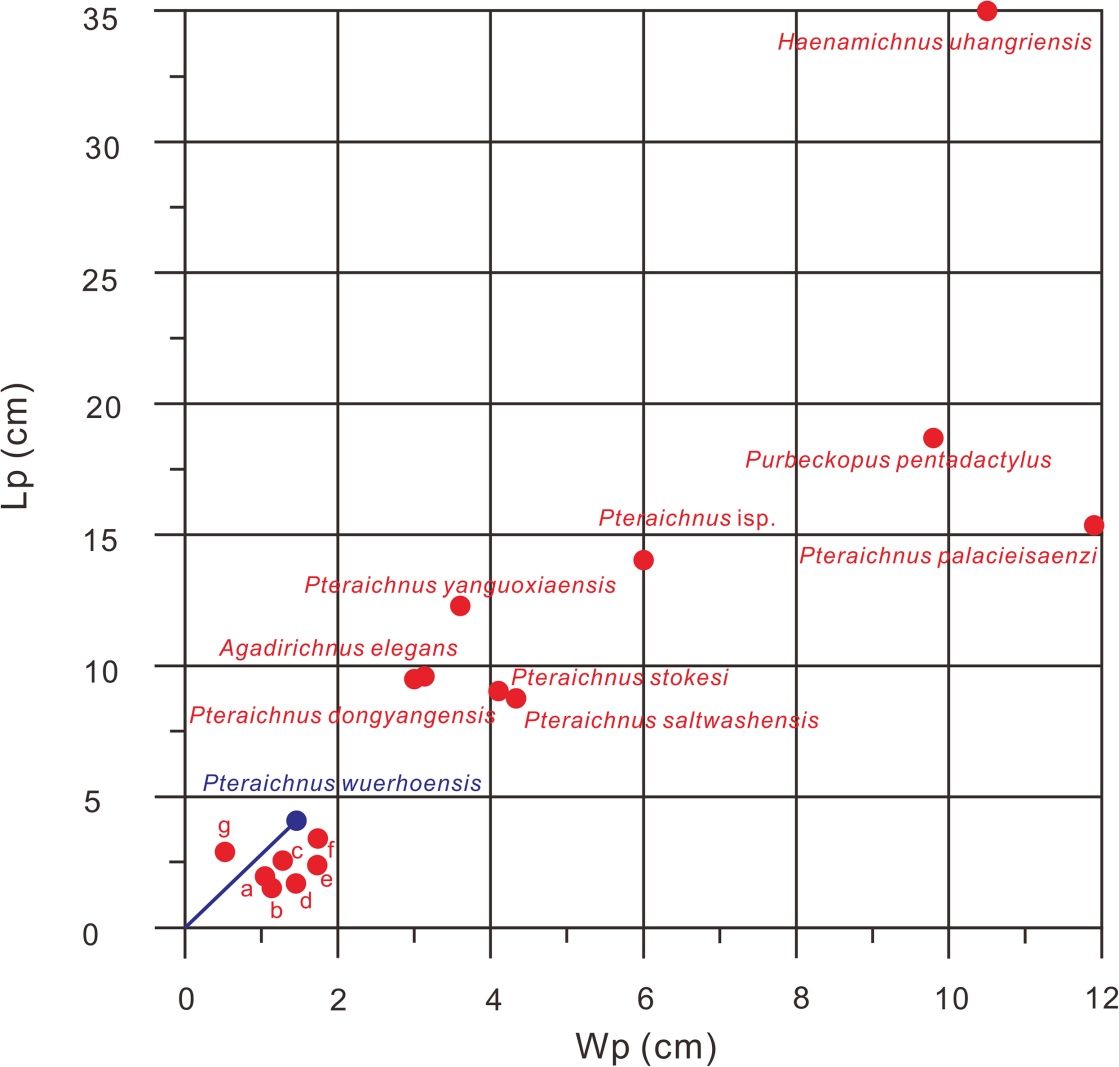
Comparison of Lp/Wp ratios of 15 ichnospecies of pterosaur tracks, *Pteraichnus wuerhoensis* isp. nov. and *Pteraichnus* isp. reported by He et al. (2013). The data comes from the original publication in which the ichnospecies were defined. a *Pteraichnus nipponensis*, b *Pteraichnus parvus*, c *Pteraichnus koreanensis*, d *Ramphichnus lafaurii*, e *Ramphichnus pereiraensis*, f *Pteraichnus longipodus*, g *Ramphichnus crayssacensis*, the slope of the blue line represents the average Lp/Wp of Pteraichnus wuerhoensis isp. nov. Abbreviations: Lp = Length of pes imprint; Wp = Width of pes imprint.

**Figure 7:**
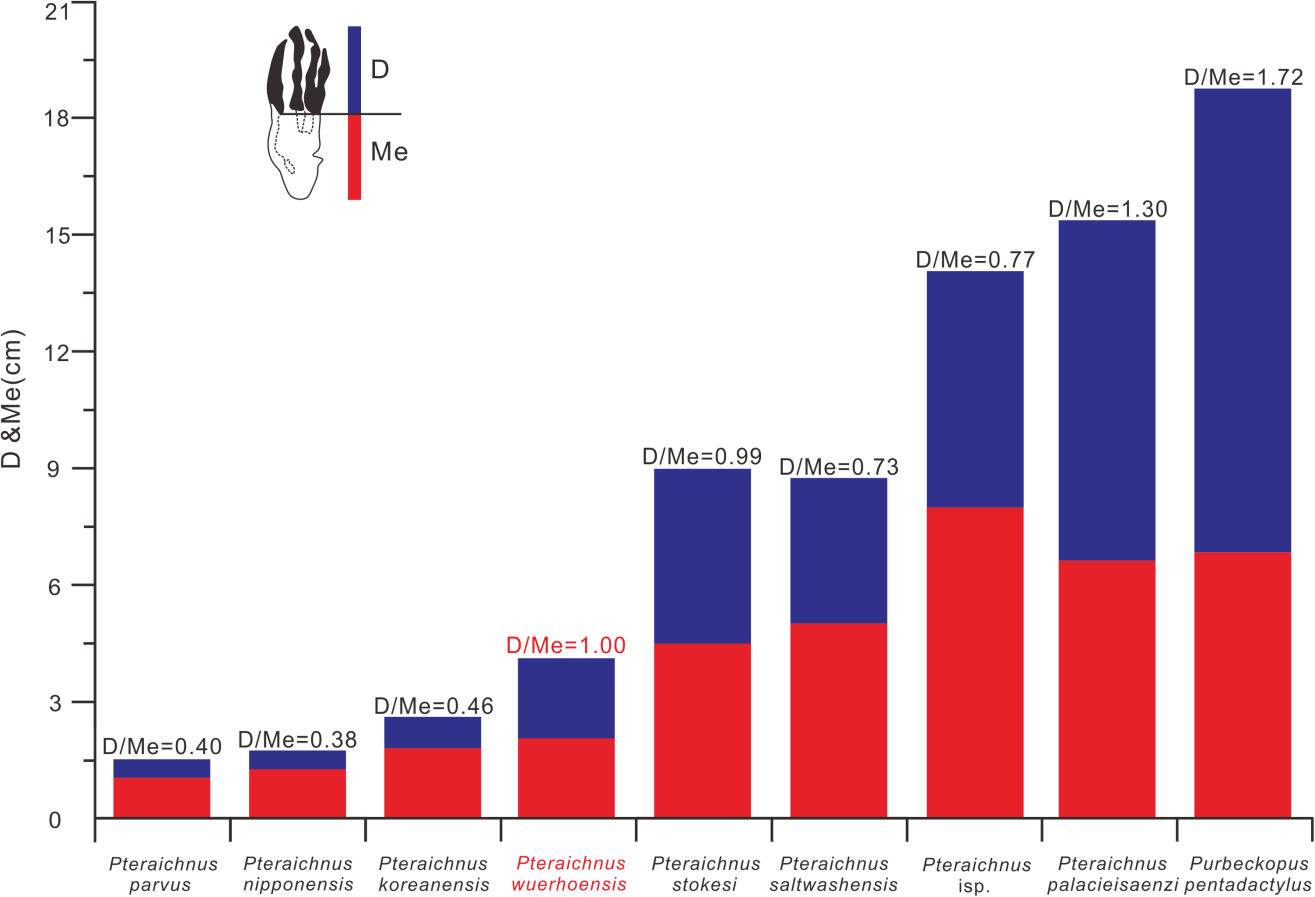
Comparison between D/Me ratios of reported ichnospecies of Pteraichnidea, *Pteraichnus wuerhoensis* isp. nov. and *Pteraichnus* isp. *Pteraichnus* isp. was reported by He et al. (2013) from the Wuerho region. Abbreviations: D = Length of digital part in pes imprint; Me = Length of metatarsal part in pes imprint.

Ichnofamily Pteraichnidae (Lockley et al., 1995) was described from a wide trackway of a quadrupedal animal with elongate, symmetrical, functional tetradactyl, plantigrade pes impressions, and an asymmetric tridactyl manus impressions. The impression of manus digit III was elongate, curved, and posteriorly directed, parallel to the trackway axis. Manus impressions often were more deeply impressed than pes impressions. The characteristics of the Wuerho small pterosaur tracks are consistent with the main features of the Pteraichnidae. To date, there are two described ichnogenera, 10 ichnospecies and more than 19 *Pteraichnus* isp., cf. *Pteraichnus*, *Pteraichnus*-like and *Purbeckopus* cf. *pentadactylus* are attributed to this family: *Pteraichnus* (Stokes, 1957), with the ichnospecies *P. saltwashensis* (Stokes, 1957); *P. stokesi* (Lockley et al., 1995); *P. parvus* (Calvo & Lockley, 2001); *P. longipodus* (Fuentes-Vidarte et al., 2004); *P. koreanensis* (Lee et al., 2009); *P. palacieisaenzi* (Pascual-Arribas & Sanz-Pérez, 2000); *P. dongyangensis* (Chen et al., 2013); *P. yanguoxiaensis* (Peng et al., 2004); *P. nipponensis* (Lee et al., 2010); and the ichnogenera *Purbeckopus* (Delair, 1963), with one ichnospecies *Purbeckopus pentadactylus* (Delair, 1963). *Purbeckopus* was first established by Delair (1963) and then was amended by Wright et al. (1997) and was maded by a quadrupedal animal with elongate (approximately twice as long as wide), subtriangular, symmetrical, functionally tetradactyl, plantigrade pes impressions; elongate, asymmetrical tridactyl manus impressions may also be present. The digits of the pes are sub-equal in length and are curved slightly inwards, the curvature being most pronounced in the outermost toe (digit IV). Digits II and III of the pes imprints are slightly longer than the outer digits I and IV. Manus impressions, if present, lie outside the pes impressions (Fig. 5G). The Wuerho small pterosaur tracks differ significantly from the *Purbeckopus* tracks in size and morphology; the latter are roughly 4.65 times the size of the former (Table 2). In addition, the pterosaur footprints from Wuerho are much more slender than those of *Purbeckopus*, and D/Me is 1.72 times as much as the Wuerho small pterosaur tracks (Table 2, Figs. 6 and 7). Ichnogenus *Pteraichnus* was first reported by Stokes (1957) and subsequently amended by Lockley et al. (1995) and Billon-Bruyat & Mazin (2003). The fossil is described as quadruped tracks with an elongate, asymmetrical, digitigrade, and tridactyl manus imprint; digit I imprint anterior or anterolateral (generally with claw mark); digit II imprint anterolateral to posterolateral (rarely with claw mark); digit III imprint posterior (exceptionally with claw mark), digital imprints increasing in length; digit IV is rarely marked and limited to the impression of the proximal part of the fourth manus digit (oriented posteromedially); a rounded impression in the medial margin of the manus imprint (impression of the fourth metacarpo-phalangeal joint); elongate, subtriangular, plantigrade, and tetradactyl pes imprint; toe II and III imprints are slightly longer than I and IV; toe I–IV imprints are clawed; manus imprints on the same axis or more laterally to the pes imprints; pes imprint anterior to the ipsilateral manus imprint; manus imprint as or more deeply impressed than pes imprint. The Wuerho small pterosaur footprints feature strongly asymmetrical, tridactyl, elongate manus imprints; digit I is the shortest and is laterally or posterolaterally oriented,; digit II is intermediate in length, longer than digit I and posterolaterally oriented; the crescent-shaped digit III is the longest and is posterolaterally oriented with a distal curvature toward the medial side and elongate, fully plantigrade, sub-triangular, tetradactyl pes imprints, with toe II and III prints longer than I and IV. Therefore, the small-sized Wuerho pterosaur tracks have similar characteristics to the features of *Pteraichnus*.

*P. saltwashensis*, which was the first reported pterosaur trackway, was described from the Upper Jurassic (Morrison) in Apache, Arizona, USA. The imprints are quite different from the Wuerho small pterosaur tracks in size and morphology. First, *P. saltwashensis* tracks are approximately 2.18 times the size of the Wuerho pterosaur tracks, where the pes length is 87.6 mm (Fig. 5H, Table 2). Second, *P. saltwashensis* (D/Me = 0.73) has a more elongate metatarsal impression than the Wuerho small pterosaur tracks (D/Me = 1.00) (Table 2, Fig. 7). Third, the Wuerho small pterosaur pes footprints (Lp/Wp = 2.75) are much more slender than those of *P. saltwashensis* (Lp/Wp = 2.02) (Fig. 6). Fourth, the medial two digits of pes imprints in Wuerho small pterosaur tracks are roughly equal to digit IV, while in *P. saltwashensis*, this is not so. Finally, the digit I imprint is often oriented laterally or posterolaterally in the Wuerho manus footprints so that the divarication between digits I and II is much smaller than that of the *P. saltwashensis* (the former is 33.5°, the latter is 43.4° Table 2). *P. stokesi* was described from the Upper Jurassic (Sundance Formation) in Alcova Lake, Wyoming, USA (Lockley et al., 1995) and was later defined as a junior synonym of *P. saltwashensis* (Billon-Bruyat & Mazin, 2003). Later, Sánchez-Hernández, Przewieslik & Benton (2009) considered it as a valid ichnospecies based on the manus (70 mm) being quite shorter than the pes (90 mm), while in *P. saltwashensis* this is on the opposite. Althougth the ratios of D/Me are similar to the Wuerho small pterosaur tracks, the pes imprints of *P. stokesi* are approximately 2.24 times the size of the Wuerho small pterosaur tracks (Fig. 5I, Table 2). The Wuerho small pterosaur tracks are much more slender than those of *P. stokesi* (Lp/Wp of fomer is 2.75, while that of *P. stokesi* is 2.20) (Fig. 6), There are six pterosaur ichnospecies (*P. palacieisaenzi*, *P. cidacoi*, *P. manueli*, *P. vetustior*, *P. longipodus and P. parvus*) referred to *Pteraichnus* reported from the Lower Cretaceous (Huérteles Allo Formation, Oncala Group) in Soria, Spain. However, according to the ICZN, due to minor differences between them and preservation conditions, four (*P. palacieisaenzi*, *P. cidacoi*, *P. manueli*, and *P. vetustior*) were reassessed as nomina nuda (Pascual-Arribas & Sanz-Pérez, 2000; Fuentes-Vidarte, 2001; Meijide-Calvo, 2001; Meijide-Fuentes, 2001; Billon-Bruyat & Mazin, 2003; Sánchez-Hernández, Przewieslik & Benton, 2009). Pascual-Arribas et al. (2014) redescribed the details of *P. palacieisaenzi* and considered it to be a valid ichnospecies. First, the pes imprint of *P. palacieisaenzi* are 3.82 times the size of the Wuerho small pterosaur tracks and show a lower Lp/Wp relative to the latter (the former is 1.29, while the latter is 2.75) (Table 2, Fig. 6). Second, the divarication between digits I–IV is 3.23 times as much as the Wuerho small pterosaur tracks (the former is 47.2°, while the latter is 14.6°). Third, the digital length of *P. palacieisaenzi* is more elongate than in the Wuerho small pterosaur tracks (the former D/Me is 1.30, while the latter is 1.00) (Fig. 7). Fourth, apart from the size difference for the manus imprints, the imprint of digit I is always anterolateral of *P. palacieisaenzi*, which makes the angle between digits I–II clearly larger than in the Wuerho small pterosaur tracks (the former is 75.9°; the latter is 33.5°) (Fig. 5J). Finally, the most significant difference between the *P. palacieisaenzi* and the Wuerho small pterosaur tracks is that the former has interdigital webbing extending from the metatarso-phalangeal joint near to the bases of the claws (Fig. 5J). *P. longipodus* tracks are slightly smaller than the Wuerho small pterosaur tracks, and Lp/Wp is lower than in the latter (Table 2). In addition, the digital length of Wuerho pes imprints is quite different from that of *P. longipodus* (the former is 16.6–21.6–20.6–19.7 mm, while the latter is 4.7–4.5–3.9–6.0 mm). *P. longipodus* has a shorter digital length than metatarsal imprint (the D/Me of *P. longipodus* is 0.22, while the D/Me of the Wuerho small pterosaur tracks is 1.00). (Fig. 7). For the manus imprints of *P. longipodus*, the divarication between digits I–II and digits II–III is approximately equal (Table 2), while the divarication angle of digits II–III is 1.74 times as much as in digits I–II for the Wuerho small pterosaur tracks (Table 2, Fig. 5K). *P. parvus* has the smallest pterosaur footprints among the four valid ichnospecies in Spain (Meijide-Calvo et al., 2004). The lengths of its holotype manus and pes imprints are 24.6 mm and 15.3 mm, respectively (Table 2). Lp/Wp and D/Me are both smaller than those of the Wuerho small pterosaur tracks (Figs. 6 and 7). This means that the Wuerho small pterosaur tracks are more slender and have a more elongate metatarsal part than *P. parvus*. Due to the direction of digit I always being laterally or anterolateral in the Wuerho small pterosaur tracks, the angle between digits I and II is obviously smaller than that of *P. parvus* (Table 2, Fig. 5L).

There are four ichnospecies of *Pteraichnus* (*P. yanguoxiaensis, P. dongyangensis, P. koreanensis*, and *P. nipponensis*) reported from Asia (Peng et al., 2004; Lee et al., 2009, 2010; Chen et al., 2013). *P. yanguoxiaensis* was described from the first reported *Pteraichnus* tracks in Asia from the Lower Cretaceous (Hekou Formation) at the Yanguoxia tracksite, Gansu, China. The tracks were quite different from the Wuerho small pterosaur tracks in size and morphology. First, the *P. yanguoxiaensis* tracks are approximately 3.06 times the size of the Wuerho small pterosaur tracks (Table 2). Second, the Lp/Wp of *P. yanguoxiaensis* is larger than that of the Wuerho small pterosaur tracks, meaning that the pes imprints of *P. yanguoxiaensis* are more slender than those of the Wuerho small pterosaur tracks (Fig. 6). Finally, the divarication angle of digits I–II is also larger than the angle of the Wuerho small pterosaur tracks (the former is 60.7°, while the latter is 33.5°) (Table 2, Fig. 5M). *P. koreanensis* was described from the Lower Cretaceous (Hasandong Formation) in Hadong County, South Korea, and was the first set of *Pteraichnus* tracks discovered in Korea. First, the Wuerho small pterosaur tracks are 1.56 times the size of *P. koreanensis*, and Lp/Wp is 1.38 times as much as the latter (the former is 2.0, the latter is 2.75) (Table 2, Fig. 6). Secondly, *P. koreanensis* has more enlongate metatarsal part than Wuerho small pterosaur tracks (D/Me for the former is 0.46, while the latter is 1.00) (Fig. 7). For the manus imprints of *P. koreanensis*, the divarication angle of digits I–II is also larger than for the Wuerho small pterosaur tracks (the former is 68.5°, while the latter is 33.5°) (Table 2, Fig. 4N). *P. nipponensis* was reported from Lower Cretaceous (Kitadani Formation) in Katsuyama City, Fukui Prefecture, Japan. The features of the anteriorly oriented digit I imprint of the manus imprints and the roughly subequal length of digits II–IV of the pes imprints are similar to the Wuerho small pterosaur tracks. However, there are differences in size and morphology. First, the Wuerho pterosaur tracks are 2.07 times the size of *P. nipponensis* and have a more elongated digital part (D/Me for the former is 1.00, while the latter is 0.38) (Table 2, Fig. 7). Second, the most significant difference between the *P. nipponensis* and Wuerho small pterosaur tracks is that the former have interdigital webbing extending from the metatarso-phalangeal joint near to the bases of the claws (Fig. 5O). *P. dongyangensis* was described from the Upper Cretaceous (Jinhua Formation) in Dongyang City, Zhejiang Province, China. According to the incorrect measurement of the width of the pes imprints, Xing et al. (2013a) and He et al. (2013) regarded ithis as *Pteraichnus* isp. However, Li (2015) re-measured the width of the pes imprints (the width is 30 mm) and regarded the specimen as a valid ichnospecies (according to the large angle between digits I–II of the manus imprints and the deeper heel impressions). Obviously, *P. dongyangensis* differs in size and morphology. First, *P. dongyangensis* is 2.24 times the size of the Wuerho small pterosaur tracks and has greater Lp/Wp ratio (Table 2, Fig. 5), meaning than the Wuerho small pterosaur tracks are more slender than those of *P. dongyangensis*. Second, for the manus imprints, the divarication angle of digits I–II is 1.55 times as much as the Wuerho small pterosaur tracks (Fig. 5P).

In addition to the nine valid ichnospecies of *Pteraichnus*, there are many other pterosaur tracks (from Upper Jurassic to Upper Cretaceous in China, Korea, France, Poland, and the USA (Figs. 5Q–5Z9)) belonging to *Pteraichnus* isp., cf. *Pteraichnus* or *Pteraichnus*-like(Lockley, 1999; Calvo & Lockley, 2001; Mazin et al., 2003; Mickelson et al., 2004; Kim et al., 2006; Fiorillo et al., 2009, 2015; Pieńkowski & Niedźwiedzki, 2005; Li, 2015; Xing et al., 2016a, 2016b; Ha et al., 2018; Masrour et al., 2018; Elgh, Pieńkowski & Niedźwiedzki, 2019). According to the sizes and morphological features, cf. *Pteraichnus* from the Later Jurassic (Summerville Formation), Ferron tracksite, central Utah, USA (Mickelson et al., 2004), is quite similar to the small-sized Wuerho pterosaur tracks (Fig. 5U). Others are quite different in size and morphology from the Wuerho small pterosaur tracks. However, due to the poor preservation conditions, cf. *Pteraichnus* lacks some significant features, and thus it is not possible to make a detailed comparison. *Pteraichnus* isp. from Upper Jurassic (Lower Kimmeridgian), Wierzbica Quarry, Poland (Elgh, Pieńkowski & Niedźwiedzki, 2019) is similar in size to the small pterosaur tracks in Huangyangquan Reservoir tracksite 1. However, the pes imprint has some major differences with the Wuerho small pterosaur tracks. First, many pes imprints of the former have obvious digit V imprints (Fig. 5Z9), while the digit V imprints are not preserved in the Wuerho small pterosaur tracks. Second, in the tracks where digit V is absent, the length of digit IV is the shortest, while the digit I is the shortest in the Wuerho small pterosaur tracks. Third, in well-preserved pes imprints of *Pteraichnus* isp. from the Wierzbica Quarry, the D/Me is larger than Wuerho small pterosaur track (Fig. 5Z9, the former is roughly 1.54, while the latter is 1.00). Finally, the trackmaker of the *Pteraichnus* isp. from the Wierzbica Quarry is likely to have been non-pterydactyloid, opposite to the trackmaker of Wuerho small pterosaur tracks. Xing et al. (2013a) and He et al. (2013) described *Pteraichnus* isp. from Tugulu Group (Lower Cretaceous) in Wuerho District, Karamay City, China, which is quite different from the Wuerho small pterosaur tracks. Xing et al. (2013a) only reported one single manus imprint that was twice the size of the Wuerho small pterosaur tracks and the divarication angle of digits I–II was 1.87 times as much as the Wuerho small pterosaur tracks (the former is 62.7°, the latter is 33.5°) (Fig. 5Z2, Table 2). He et al. (2013) described one manus-pes set in the Wuerho area that was 3.49 times the size of the Wuerho small pterosaur tracks. The pes imprint has a longer metatarsal imprint than that of the Wuerho small pterosaur tracks (D/Me of the former is 0.77, while the latter is 1.00, Fig. 7). In addition, the medial two digital lengths of *Pteraichnus* isp. reported by He et al. (2013) are longer than the lengths of the external digits, while the lengths of digits II–IV are roughly equal in the Wuerho small pterosaur tracks (Fig. 5Z1). Apart from this, the pes imprints of the Wuerho small pterosaur tracks are more slender than those of *Pteraichnus* isp. (Lp/Wp of the former is 2.75, while the latter is 2.33; Table 2, Fig. 6). For the manus imprints, in addition to the larger size, the divarication angle of digits I–II is 1.71 times as much as the Wuerho small pterosaur tracks (Table 2, Fig. 5Z1). Therefore, the small size (average Lp = 4.02 cm, Lm = 3.40 cm), elongated pes imprints (Lp/Wp = 2.75), roughly equal digits II–IV, approximately equal digital length and metatarsal part and generally laterally or posterolaterally oriented digit I of the manus imprints distinguish the Wuerho small pterosaur tracks from other reported pterosaur tracks. Therefore, we established a new ichnospecies of *Pteraichnus wuerhoensis* isp. nov.

### Evidence for the trackmaker of *Pteraichnus wuerhoensis* isp. nov.

**Figure 8:**
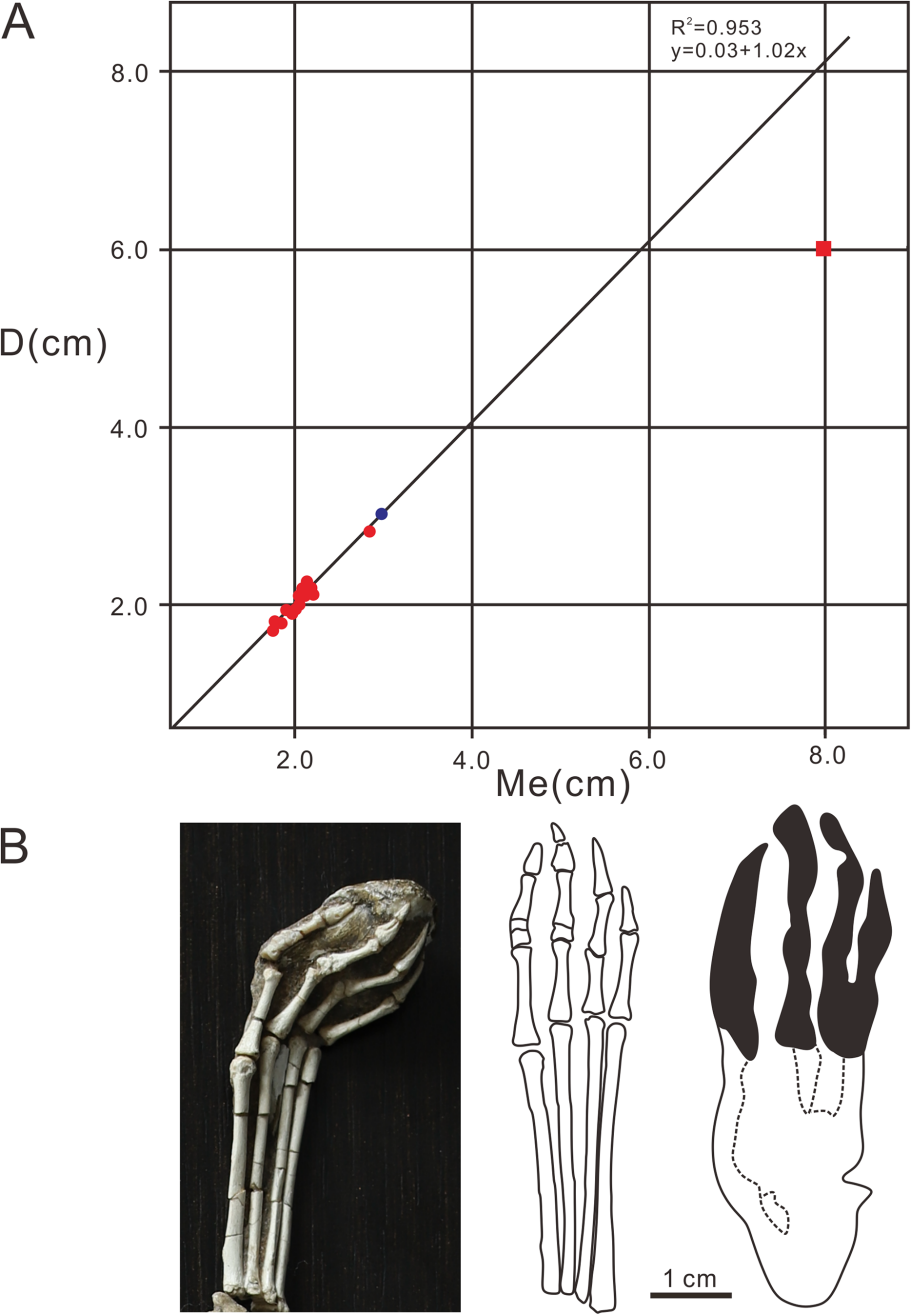
Comparative analysis diagrams of *Pteraichns wuerhoensis* isp. nov. and pes bone fossil of *N. complicidens*. (A) Scatter plot and linear fitting curve (black line) for D and Me values, the red dots represent the D and Me values of the pes imprints, the bule dot represents the value of pes bone fossil, the red square represents the D and Me of *Pteraichnus* isp. reported by He et al. (2013); (B) the pes bone fossil, outline drawing and pes imprint of *N. complicidens*. Abbreviations: D = Length of digital part in pes imprint; Me = Length of metatarsal part in pes imprint.

The pterodactyloid and non-pterodactyloid pterosaurs have well-known differences in the morphology of the pedes (Lockley, Harris & Mitchell, 2008). The latter has a unique pleiomorphic elongate but very delicate digit V (Wang et al., 2002, 2009, 2010; Jiang et al., 2015). Generally speaking, of all the vertebrate taxa, the Pterosauria are the only clade featured by tridactyl manus imprints located laterally or posterolaterally to the pes imprints, meaning that the width of the manus imprints trackway is wider than the pes imprints trackway (Mazin & Pouech, 2020). Lockley, Harris & Mitchell (2008) questioned whether the delicate digit V in non-pterodactyloids would register in pes footprints. The reason for this uncertainty is that almost all the pterosaur footprints reported in the world are similar in shape (strongly asymmetrical manus imprints, elongate, fully plantigrade, tetradactyl pes imprints, manus imprints located laterally or posterolaterally to the pes imprints, and much wider trackways of manus imprints). However, with the discovery of identified non-pterodactyloid footprints in France (Mazin & Pouech, 2020), there are several features that can be used to discriminate pterodactyloid from non-pterodactyloid footprints. The non-pterodactyloid footprints are characterized by fully plantigrade or digitigrade, pes imprints and tridactyl, digitigrade, non-rotated manus imprints. The manus trackway is wider than, or equal to the pes trackway (Mazin & Pouech, 2020). Therefore, based on the morphological features of the Wuerho small pterosaur tracks, we inferred that the trackmaker of the Wuerho pterosaur small footprints was a pterodactyloid (all the manus imprints are strongly asymmetrical and pes imprints are tetradactyl, fully plantigrade). All known Cretaceous pterosaurs are pterodactyloid. The age of the Wuerho small pterosaur tracks is the Early Cretaceous, which is consistent with the age of pterodactyloids. A footprint is not only related to the autopod anatomy, but also to the soft tissues (surrounding the bones), substrate, stance, gait and velocity of the trackmaker (Baird, 1980). Therefore an imprint is the result of the dynamic interaction between the autopod and the substrate (Baird, 1980). There are two useful methods commonly used to identify the trackmaker. One is to reconstruct the autopod anatomy characteristics from footprints and then compare the result to those of the potential trackmakers in the fossil record (Billon-Bruyat & Mazin, 2003). The other method is just the reverse (Unwin, 1989). However, Billon-Bruyat & Mazin (2003) stated that the theoretical footprints obtained by the second method are generally smaller than the natural footprints. Therefore, we adopted the first method to obtain some skeletal characteristics from the Wuerho small pterosaur footprints and compared the results with the pterosaur fossils in the Junggar Basin to accurately identify the trackmaker. To date, the records of the Early Cretaceous pterosaur osteological fossils in Wuerho region, northwest Junggar Basin include one family, two genera and two species: Dsungaripteridae (*D. weii, N. complicidens*) (Young, 1964, 1973). *Dsungaripterus weii* was first reported by Young (1964) from the Lower Cretaceous (the Hutubihe Formation, Shengjinkou Formation and Lianmuqin Formation have all yielded fossils) in the Wuerho area, Xinjiang, China, and was the first relatively complete pterosaur skeleton from China. The fossil featured a rather large size, and the total width of the wings was presumably between 3–3.5 m. However, there are no manus or pedes bone fossils from the known material (Young, 1964). *N. complicidens* was discovered from the same locality and horizon and was an adult individual at least one third smaller in size than *D. weii* (Young, 1973). Furthermore, there are well-preserved manus and pedes bone fossils in the paratype (IVPP RV 73001). To date, two types of *Pteraichnus* isp. have been reported from the Wuerho region, and both are quite different from the Wuerho small pterosaur tracks in size and morphology. Xing et al. (2013a) only reported a single manus imprint that was 2 times the size of the Wuerho small pterosaur tracks and the divarication angle of digit I–II is 1.87 times as much as the angle of the Wuerho small pterosaur tracks (the former is 62.7°, while the latter is 33.5°) (Fig. 5Z2, Table 2). He et al. (2013) described one manus-pes set in Wuerho area, which is 3.49 times the size of the Wuerho small pterosaur tracks. For the pes, there is a longer metatarsal imprint in the Wuerho small pterosaur tracks (D/Me of the former is 0.77, while the latter is 1.00, Fig. 7). In addition, the media two digital lengths of *Pteraichnus* isp. reported by He et al. (2013) are longer than the length of the external digits, while the lengths of digits II–IV are roughly equal in the Wuerho small pterosaur tracks (Fig. 5Z1). Apart from this, the pes imprints of the Wuerho small pterosaur tracks are more slender than those of *Pteraichnus* isp. (Lp/Wp of the former is 2.75, while the latter is 2.33; Table 2, Fig. 6). For the manus imprints, in addition to the larger size, the divarication angle of digits I–II is 1.71 times the size of the angle of the Wuerho small pterosaur tracks (Table 2, Fig. 5Z1). Therefore, according to the obvious differences between the *Pteraichnus* isp. and Wuerho small pterosaur tracks, we inferred that the Wuerho small pterosaur footprints and the *Pteraichnus* isp. in the Wuerho area were left by at least two kinds of pterosaurs with different sizes. In addition, the manus imprints described by Xing et al. (2013a) and He et al. (2013) have similar characteristics (large size, anterolaterally oriented digit I). Therefore, we tentatively speculate that the trackmakers of *Pteraichnus* isp. in Wuerho area may be the same kind of pterosaur. Based on the two different sizes and forms of pterosaur tracks from two different sizes of pterosaurs in the same horizon in the Wuerho area, we inferred that the trackmaker of the large pterosaur tracks may be *D. weii*, while the trackmaker of the Wuerho small pterosaur tracks may be *N. complicidens*. To further corroborate our conjecture, we extracted the main features (digits length of pes imprint, D, Me, D/Me) of the Wuerho small pterosaur footprints and compared them with the pes bone fossils of *N. complicidens*. We did not choose the measurement parameters of the manus imprint, because the characteristics of pterodactyl manus imprints are very similar and the commonly used measurement methods are not consistent with the actual length of the bone fossils or the proportional relationship between them. Through the comparison with the pes bone fossil of *N. complicidens* (Table 1), we discovered two main features that were consistent with *N. complicidens*. One was the relative length of digits I–IV (Table 1, digit I imprint is the shortest and other three digits are roughly subequal in length). The other was that the ratio of D/Me of the pes bone fossil is consistent with the Wuerho pterosaur tracks (Table 1, Fig. 8, *n* = 15). According to the scatter plot (Fig. 8), the D and Me values of pes bone fossils fall on the linear regression line. The D/Me value of the large pterosaur pes imprints reported by He et al. (2013) is below the linear regression line (Fig. 8). Therefore, according to the features of the Wuerho small pterosaur tracks, we can first infer that the footprints were left by small pterosaurs. Second, there is only one species of small pterosaur (*N. complicidens*) in the Wuerho area, and it is mainly distributed in the Shengjinkou formation, corresponding to the horizon of the Wuerho small pterosaur tracks. Third, the anatomical features extracted from the Wuerho small pterosaur pes imprints are consistent with the pes bone fossil of *N. complicidens*. Fourth, in terms of size, the two different sizes of pterosaur tracks in Wuerho area are consistent with the records of pterosaurs bone fossils. These lines of evidence suggest that the trackmaker of the Wuerho small pterosaur footprints probably was *N. complicidens* and the total width of the wings was presumably between 2 and 2.3 m (Young, 1973). The large pterosaur tracks described by Xing et al. (2013a) and He et al. (2013) may have been left by *D. weii* and the total width of the wings was presumably between 3–3.5 m

### Some possible behaviors of the trackmaker

There were 57 manus imprints and 57 pes imprints preserved on the same slab (Fig. 2, 125 cm × 25 cm), meaning that the trackmaker was possibly quadrupedal. Although we cannot conclude that these footprints were left at the same time, they were certainly formed within a short period time. This is because when the footprints were left, they would have been quickly buried by sediments and then eventually have become the fossilized (Lockley & Hunt, 1995; Li, 2015). Otherwise, they would have been destroyed by waves or other geological agents (Lockley & Hunt, 1995; Li, 2015). In addition, the Wuerho small pterosaur tracks vary in size (Table 1) (the size of manus imprints ranges from 1.90 to 5.15 cm, while the pes imprints range from 2.68 to 5.71 cm), suggesting that these footprints were left by pterosaurs of different ages. The density of the Wuerho small pterosaur tracks is also high (approximately 365/m^2^). These two lines of evidence suggest that many pterosaurs of different ages lived at Huangyangquan Reservoir tracksite 1. Generally, high densities of tracks (in excess of 100 per m^2^) have often been cited as evidence of gregarious behavior or high activity levels (Lockley et al., 1992; Mickelson et al., 2004).

**Figure 9:**
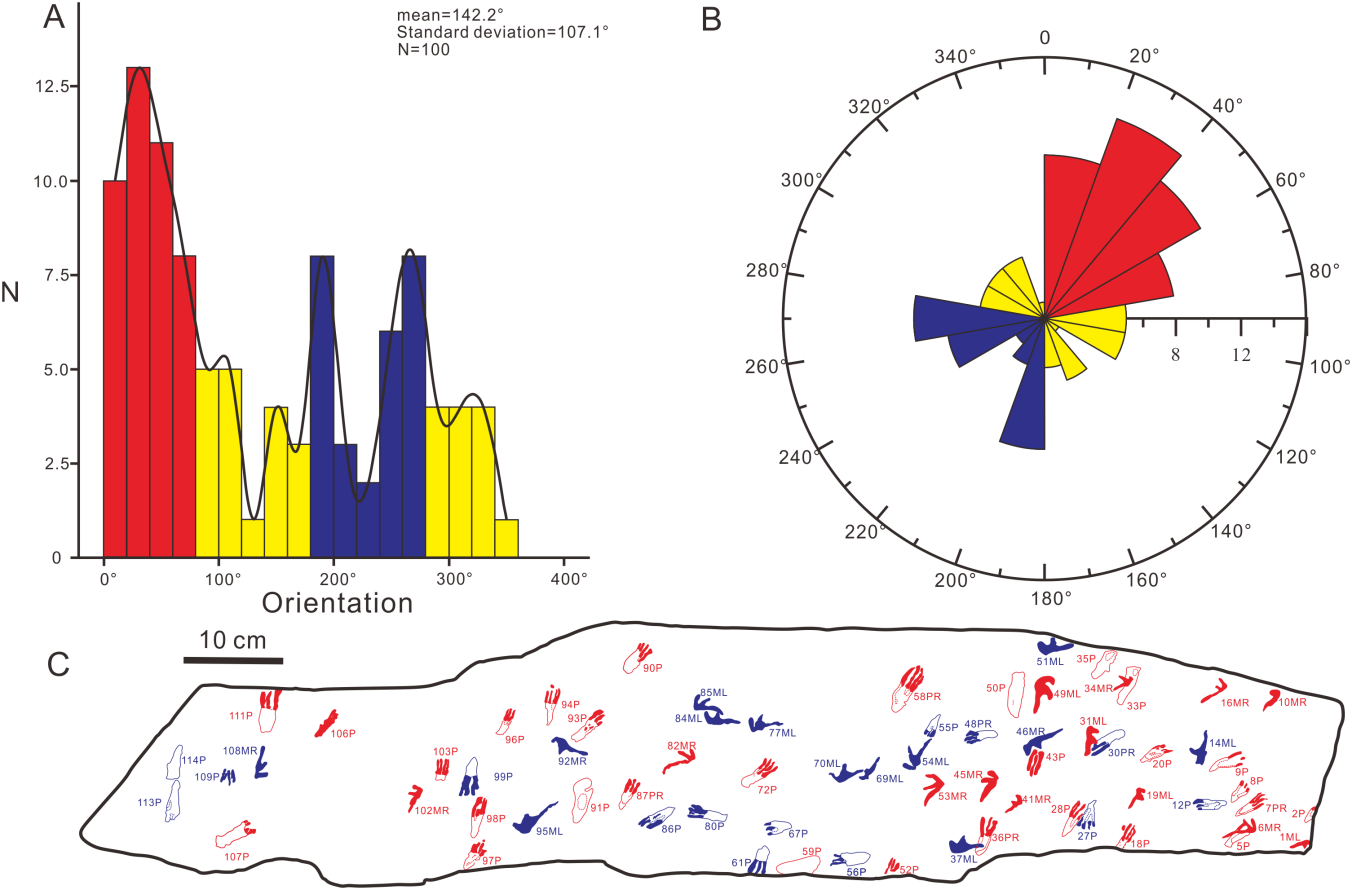
The histogram and rose diagram of the forward orientations of *Pterachnus wuerhoensis* isp. nov. (A)–(B) The histogram and the rose diagram of the forward orientations, the red color and blue color represent the two main concentrate orientations, the yellow color represents other orientations; (C) the outline drawings of footprints in two main orientations, the red color represents the footprints in orientation of 0°–80° and the blue color represents the footprints in orientation of 180°–280°. Abbreviations: P = Pes imprint; ML = Left imprint of manus; MR = Right imprint of manus; PR=Right imprint of pes.

In order to further explore the probable behavior of the trackmaker, we conducted a statistical analysis of the forward orientations of the Wuerho pterosaur tracks. We drew a rose diagram and histogram to reflect the degrees of dispersion and aggregation of the data. According to the histogram (Fig. 9A) and rose diagram (Fig. 9B), the pterosaur tracks were relatively concentrated in two opposite orientations (Figs. 9B, 9C; 0°–80°, 180°–280°). In addition, the two main opposite orientations of the footprints are oblique to the long axis of the slab, and thus we can exclude the impact of the long axis direction of the slab on the statistical results. From the analysis results, the trackmakers mainly walked in two opposite orientations, suggesting that the trackmakers may have been walking back and forth in two opposite orientations. However, we cannot exclude other possibilities. Due to the narrow slab width (25 cm) and disorderly distributed pterosaur tracks, we only recognized several possible but uncertain trackways in these two opposite orientations (Fig. 3). In addition, there are no ripple marks, invertebrate traces or turning tracks on this slab, and thus we cannot explain for this phenomenon at present. However, we believe that new pterosaur footprints will be found in Huangyangquan Reservoir tracksite 1, and we expect to be able to propose a reasonable explanation for this phenomenon.

## Conclusions

The Wuerho region has provided the largest number of pterosaur tracks currently known from China. The horizon of the Wuerho small pterosaur tracks belongs to the Lower Cretaceous Shengjinkou Formation and comprises 57 manus imprints and 57 pes imprints. According to the detailed comparison with the reported ichnospecies, we infer that the Wuerho small pterosaur tracks belong to *Pteraichnus* and are different from the 15 reported valid ichnospecies. Therefore, we proposed a new ichnospecies of *Pteraichnus wuerhoensis* isp. nov. According to the main features (digits length of pes imprint, D, Me, D/Me) of the *P. wuerhoensis *and comparisons with the pes bone fossils of *N. complicidens*. We infer that the trackmaker of *P. wuerhoensis* probably was *N. complicidens* and the total width of the wings was presumably 2–2.3 m. The high density (365 per square meter) and varied sizes of the Wuerho small pterosaur tracks suggest that many pterosaurs of different ages lived in Huangyangquan Reservoir tracksite 1. This implies the trackmaker of *P. wuerhoensis* may have gregarious behavior.

## Institutional Abbreviations

IVPP: Institute of Vertebrate Paleontology and Paleoanthropology
MGCM: Moguicheng Dinosaur and Bizarre Stone Museum
ICZN: International Commission on Zoological Nomenclature

## Additional Information and Declarations

### Data Availability

The following information was supplied regarding data availability: We use photogrammetry and Agisoft PhotoScan Professional to establish three-dimensional model. The raw data of the 3D model, 3D Project and original photos is available at MorphoSource DOI 10.17602/M2/M346640. The accession number of this specimen is IVPP V 26281.2 and is kept in IVPP (Institute of Vertebrate Paleontology and Paleoanthropology), Beijing, China.

### New Species Registration

The following information was supplied regarding the registration of a newly described species: Publication LSID: urn:lsid:zoobank.org:pub:DF34F23C-8F5A-4CA7-AF42-EB95961E6418 Species name: *Pteraichnus wuerhoensis* LSID: urn:lsid:zoobank.org:act:D4FCD320-07CA-4601-B001-0FDEA0070532.
